# Inhibition of the ILK-AKT pathway by upregulation of PARVB contributes to the cochlear cell death in *Fascin2* gene knockout mice

**DOI:** 10.1038/s41420-024-01851-5

**Published:** 2024-02-19

**Authors:** Rongrong Liu, Wenjing Shang, Yingying Liu, Yi Xie, Jun Luan, Ting Zhang, Ying Ma, Zengxian Wang, Yan Sun, Xicheng Song, Fengchan Han

**Affiliations:** 1 Department of Biochemistry and Molecular Biology, and Key Laboratory for Genetic Hearing Disorders in Shandong, Binzhou Medical University, 346 Guanhai Road, Yantai, 264003 Shandong PR China; 2 Department of Otorhinolaryngology-Head and Neck Surgery, Affiliated Yantai Yuhuangding Hospital of Qingdao University, Yantai, 264000 PR China; 3 Institute of Neurobiology, School of Medicine, Xi’an Siyuan University, 28 Shui An Road, Xi’an, 710038 Shaanxi PR China

**Keywords:** Cellular neuroscience, Cochlea

## Abstract

The *Fscn2* (*Fascin2*) gene encodes an actin cross-linking protein that is involved in the formation of hair cell stereocilia and retina structure. Mutations in *Fscn2* gene have been linked to hearing impairment and retinal degeneration in humans and mice. To understand the function of the *Fscn2* gene, we generated the *Fscn2* knockout mice, which showed progressive loss of hearing and hair cells. Our goal of the present study was to investigate the mechanism underlying cochlear cell death in the *Fscn2* knockout mice. Microarray analysis revealed upregulation of expression of PARVB, a local adhesion protein, in the inner ears of *Fscn2* knockout mice at 8 weeks of age. Further studies showed increased levels of PARVB together with cleaved-Caspase9 and decreased levels of ILK, p-ILK, p-AKT, and Bcl-2 in the inner ears of *Fscn2* knockout mice of the same age. Knockdown of *Fscn2* in HEI-OCI cells led to decreased cell proliferation ability and migration rate, along with increased levels of PARVB and decreased levels of ILK, p-ILK, p-AKT, Bcl-2 and activated Rac1 and Cdc42. Overexpression of *Fscn2* or inhibition of *Parvb* expression in HEI-OC1 cells promoted cell proliferation and migration, with increased levels of ILK, p-ILK, p-AKT, and Bcl-2. Finally, FSCN2 binds with PPAR-γ to reduce its nuclear translocation in HEI-OC1 cells, and inhibition of PPAR-γ by GW9662 decreased the level of PARVB and increased the levels of p-AKT, p-ILK, and Bcl-2. Our results suggest that FSCN2 negatively regulates PARVB expression by inhibiting the entry of PPAR-γ into the cell nucleus, resulting in inhibition of ILK-AKT related pathways and of cochlear cell survival in *Fscn2* knockout mice. Our findings provide new insights and ideas for the prevention and treatment of genetic hearing loss.

## Introduction

Hearing loss is a significant health concern that affects more than 5% of the global population [1, 2]. Despite the identification of over 120 deafness genes, developing effective treatments for hereditary deafness remains a challenge due to the lack of understanding of the molecular and cytological behaviors underlying the condition [3]. Mouse models have been proven to be the ideal tools for studying deafness-related genes due to the similarities between mice and humans in terms of genetic structure, anatomy, and pathological features [4, 5].

Johnson KR et al. found that an *ahl8* locus was associated with the progressive hearing loss in DBA/2J mice by genetic linkage analysis [6]. Later, they confirmed that the *ahl8* locus was a missense mutation (p.R109H) in *Fascin-2* (*Fscn2*) gene [7]. FSCN2 is an actin cross-linking protein, which can bind to a variety of actin (α-,β-,γ-actin) [8, 9] and participate in the formation of stereocilia of mouse and retina of human [10]. The crosslinking function of FSCN2 is mainly reflected in slowing down the depolymerization of actin at the top of stereocilia and maintaining the length of stereocilia and the auditory function of mice [9]. Mutations in the *Fscn2* gene can lead to hearing impairment in mice and retinal degeneration in humans [7, 11–13]. Our previous study found that cochlear cell apoptosis was associated with hearing impairment in DBA/2 J mice, and anti-apoptotic agents could delay the hearing loss to some extent [14]. However, this therapy could not change the outcome of deafness in mice, and further research is necessary to understand the mechanism of hearing loss.

To shed light on the function of *Fscn2* gene, we used TALEN technology [15] to knockout *Fscn2* in C57BL/6J mice. Although 21 amino acid residues at the amino terminus of the FSCN2 remain, it is functionally deficient. The *Fscn2* knockout (*Fscn2*^*-/-*^) mice showed progressive hearing loss starting at 3 weeks after birth, making it a new hereditary deafness mouse model [16]. In addition, F-actin staining and scanning electron microscopy (SEM) showed severe hair cell degeneration and loss of stereocilia in *Fscn2*^*−/−*^ mice. The Fascin protein has two forms, nonphosphorylated and phosphorylated (such as at position S39). Phosphorylated Fascin is involved in filopodia formation and cell migration, but its binding ability to actin is reduced [10]. FSCN2 residual peptide in *Fscn2*^*-/-*^ mouse lacks both actin-binding sites and phosphorylation sites, affecting the migration function of cells.

Local adhesion or focal adhesion, as a mechanical connection and biological signaling center between cells and the external environment, forming and guiding many signaling related proteins at the site of integrin binding [17–19]. As a class of local adhesion proteins, Parvins protein family in mammals has three members, α-, β- and γ-Parvin, which are encoded by three different genes, *Parva, Parvb* and *Parvg*, respectively [20, 21]. β-Parvin (PARVB) protein is localized in focal adhesion and plays an important role in cell adhesion, proliferation, and migration by activating multiple signaling pathways [22]. PARVB is expressed in a variety of mammalian tissues and cells, and it functions by interacting with ILK, α-PIX and α-actinin, and participates in signal transduction pathways that regulate actin cytoskeleton dynamics and cell survival. It plays a crucial role in the occurrence and development of many human diseases [23–25].

In this study, we aimed to explore the molecular mechanism of hearing loss in *Fscn2* knockout mice. We found that downregulation of the ILK-AKT-Bcl2 related pathways by upregulation of PARVB, which results from loss of inhibition of FSCN2 to PPAR-γ, was involved in the degeneration of cochlear cells in *Fscn2*^*−/−*^ mice. Our results would provide new insight into the prevention and treatment of hereditary deafness.

## Results

### The densities of spiral ganglion neurons in *Fscn2*^*−/−*^ mice were reduced progressively over time

**Fig. 1:**
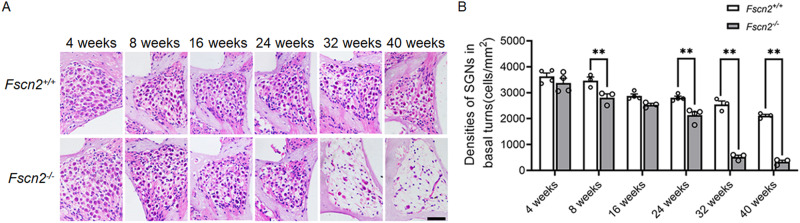
The densities of spiral ganglion neurons (SGNs) in the cochleae of *Fscn2*^*−/−*^ mice were gradually decreased. **A** SGN densities were detected in *Fscn2*^*+/+*^ mice and *Fscn2*^*−/−*^ mice by HE staining. **B** The densitometric analysis was made by ImageJ software. Data are represented as means ± SEM. Scale bar, 50 μm. **P < 0.01.

*Fscn2*^*−/−*^ mice exhibited progressive hearing loss and hair cell death, as demonstrated by auditory brainstem response (ABR) tests and scanning electron microscopy in our previous study [16]. The densities of spiral ganglion neurons (SGNs) in the cochlear basal turns of *Fscn2*^*+/+*^ and *Fscn2*^*−/−*^ mice were further assessed over time using HE staining in this study. The SGN densities in *Fscn2*^*−/−*^ mice were decreased gradually from 8 weeks of age and were significantly lower than the SGN densities in *Fscn2*^*+/+*^ mice from 24 weeks of age (Fig. 1A). At 32 weeks of age, the SGN density in *Fscn2*^*−/−*^ mice was reduced by 84.5% compared to 4 weeks of age, and was decreased by 78.6% compared to *Fscn2*^*+/+*^ mice of the same age. By 40 weeks of age, *Fscn2*^*−/−*^ mice had only 10% of the SGNs remaining, a reduction of 85.3% compared to *Fscn2*^*+/+*^ mice of the same age (Fig. 1B).

### PARVB was upregulated in the inner ears of *Fscn2*^*−/−*^ mice

**Fig. 2:**
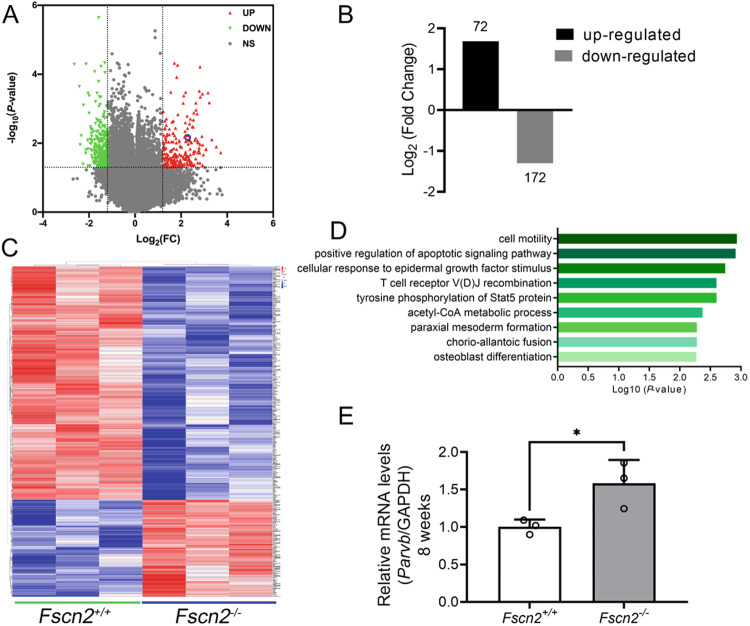
PARVB expression in the inner ears of *Fscn2*^*−/−*^ mice was upregulated. **A**, **B** Volcano plot for differentially expressed genes between the inner ears of *Fscn2*^*+/+*^ and *Fscn2*^*−/−*^ mice with age of 8 weeks. **C**, **D** Heatmap showing the significant differentially expressed genes between *Fscn2*^*+/+*^ and *Fscn2*^*−/−*^ (**C**) and the functional enrichment of the genes by GO (Gene Ontology) analysis (**D**). **E** qRT-PCR results to verify the transcription level of *Parvb* in the inner ears of *Fscn2*^*−/−*^ mice at 8 weeks of age. Data are represented as means ± SEM. *P < 0.05.

**Fig. 3:**
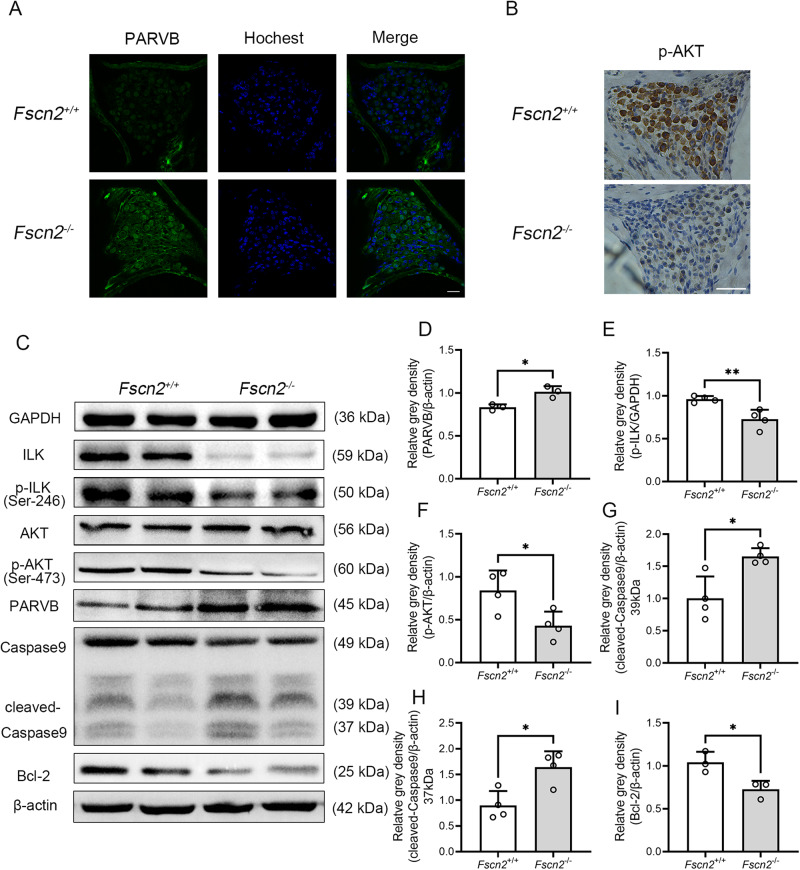
PARVB expression was upregulated, while the ILK-AKT related pathways were downregulated in the inner ears of *Fscn2*^*−/−*^ mice at 8 weeks of age. **A** The expression of PARVB in the spiral ganglion neurons (SGNs) in the cochleae of *Fscn2*^*+/+*^ and *Fscn2*^*−/−*^ mice was localized by immunofluorescence histochemical analysis. Scale bar, 20 μm. **B** The expression of p-AKT was detected by immunohistochemical staining in the cochleae of *Fscn2*^*+/+*^ and *Fscn2*^*−/−*^mice. The images are representative of three experiments. Scale bar, 50 μm. **C** The levels of PARVB, ILK, p-ILK, AKT, p-AKT, cleaved-Caspase9 and Bcl-2 were detected by Western blot in the inner ears of *Fscn2*^*+/+*^ and *Fscn2*^*−/−*^ mice at 8 weeks of age. **D**–**I** The six panels on the right side show the results of densitometric analysis of the bands on Western blots. Data are represented as means ± SEM. *P < 0.05; **P < 0.01.

Gene expression profiles in the inner ears were compared between *Fscn2*^*+/+*^ and *Fscn2*^*−/−*^ mice at 8 weeks of age using microarray analysis (GSE252226). Hearing screening tests at 8 weeks of age showed that the auditory brainstem response thresholds of *Fscn2*^*−/−*^ mice were increased to 70–75 dB SPL at stimuli of click, while those of *Fscn2*^*+/+*^ mice were within the normal range (<55 dB SPL). Microarray analysis identified a total of 244 differentially expressed mRNAs, including 72 upregulated and 172 downregulated genes (Fig. 2A, B). Gene ontology analysis revealed that the top two biological processes affected were cell movement and migration, and cell apoptotic signaling pathways (Fig. 2C, D). qRT-PCR and Western blotting confirmed that the expression level of *Parvb* gene was upregulated in the inner ears of *Fscn2*^*−/−*^ mice at 8 weeks of age (Figs. 2E, 3C, D).

### Expression of PARVB related proteins in the inner ears of *Fscn2*^*−/−*^mice

*Fscn2*^*+/+*^ mice and *Fscn2*^*−/−*^ mice at 8 weeks of age were used for detecting the expression of PARVB related proteins in the inner ears. Mouse hearing was tested as described previously [16]. The results showed that ABR (auditory-evoked brainstem response) thresholds were higher in *Fscn2*^*−/−*^ mice than in *Fscn2*^*+/+*^ mice at stimulus frequencies of 8, 16 and 32 kHz, while DPOAE (distortion product otoacoustic emission) amplitudes were lower in *Fscn2*^*−/−*^ mice than in *Fscn2*^*+/+*^ mice at f2 frequencies of 11.133 kHz and 15.741 kHz (Fig. S1B, E, Supplemental Material-1). Immunofluorescence histochemical analysis showed stronger staining of PARVB in SGNs in the cochleae of *Fscn2*^*−/−*^ mice (Fig. 3A), and immunohistochemical analysis revealed weaker staining of p-AKT in SGNs (mainly in the cytoplasm) of *Fscn2*^*−/−*^ mice (Fig. 3B), when compared to *Fscn2*^*+/+*^ mice. Western blot analysis of inner ears showed higher levels of PARVB and cleaved-caspase9, but lower levels of ILK, p-ILK, AKT, p-AKT and Bcl-2 in *Fscn2*^*−/−*^ mice, compared to *Fscn2*^*+/+*^ mice (Fig. 3C–I). Similar results were obtained in mice at 4 and 12 weeks of age Supplemental Material-1 (Figs. S1A, C, D, F; S2; S3). The results suggest that upregulation of PARVB is relevant to proteins in the ILK-AKT-Bcl-2 related pathways in the inner ears of *Fscn2*^*−/−*^ mice.

### Knockdown of *Fscn2* in HEI-OC1 cells resulted in decreased cell proliferation ability and migration rate

**Fig. 4:**
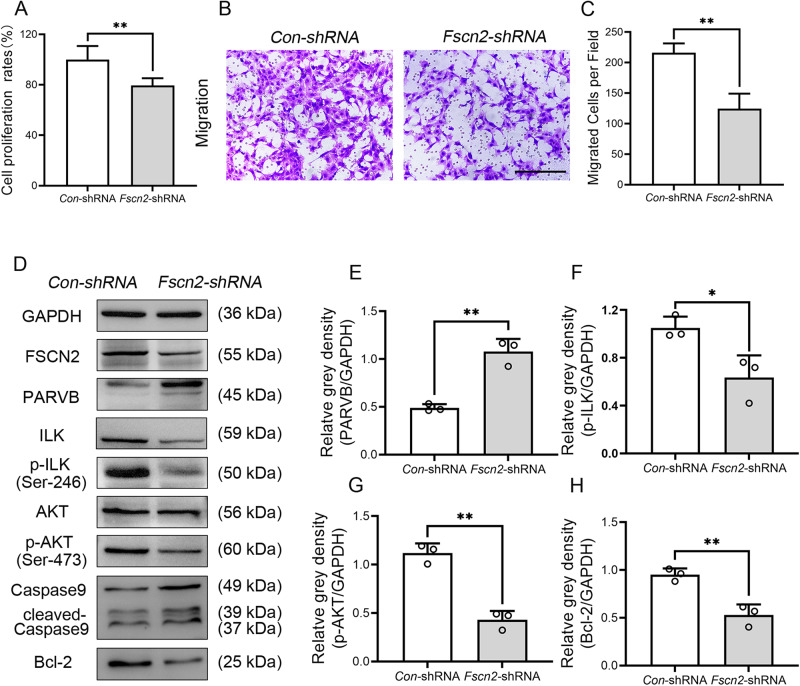
Knockdown of *Fscn2* in HEI-OCI cells resulted in low cell proliferation ability and migration rate. **A** Cell viability was assessed by CCK-8 assays. **B**, **C** The migration assay was performed to evaluate the migration ability of cells. Scale bar, 100 μm. **D** The levels of PARVB, ILK, p-ILK, AKT, p-AKT, cleaved Caspase9 and Bcl-2 in the *Fscn2* knockdown cells were detected by Western blot. **E**–**H** The four panels on the right side show the results of densitometric analysis of the bands on Western blots. Data are represented as means ± SEM. *P < 0.05; **P < 0.01.

HEI-OC1 cells with low level expression of the *Fscn2* gene were created by infection with lentiviral particles expressing *Fscn2*-shRNA. These cells showed significantly reduced cell proliferation and migration rates compared to cells infected with *Con*-shRNA (Fig. 4A–C). Western blot analysis revealed higher levels of PARVB and cleaved-caspase9, and lower levels of ILK, p-ILK, p-AKT and Bcl-2 in *Fscn2*-shRNA infected cells (Fig. 4D–H). These results suggest that low level expression of *Fscn2* leads to higher level of PARVB and lower levels of p-AKT and Bcl-2, resulting in reduced cell proliferation and migration rates in HEI-OC1 cells. The results indicate that downregulation of *Fscn2* expression reduces p-AKT-mediated proliferation ability of HEI-OC1 cells.

### Overexpression of *Fscn2* and knockdown of *Parvb* expression in HEI-OC1 cells increased cell proliferation ability

**Fig. 5:**
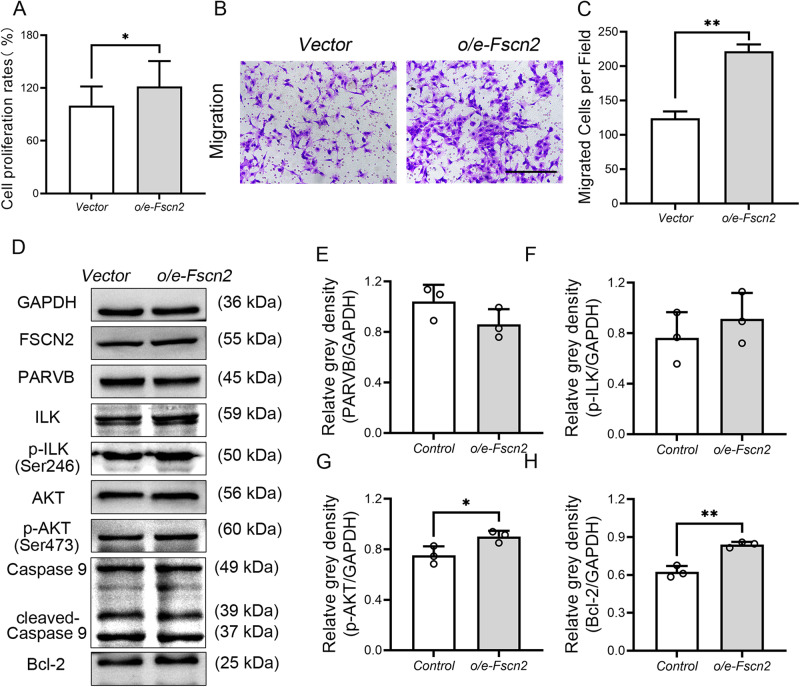
Overexpression of *Fscn2* in HEI-OC1 cells (*o/e-Fscn2* cells) lead to increment of cell proliferation ability and cell migration rate. **A** Cell viability was assessed by CCK-8 assays. **B**, **C** The migration assay was performed to evaluate the migration ability of cells. **D** The levels of PARVB, ILK, p-ILK, AKT, p-AKT, cleaved Caspase9 and Bcl-2 in *Fscn2* overexpression cell were tested by Western blot. **E**–**H** The four panels on the right side are the results of densitometric analysis of the bands on Western blots. Data are represented as means ± SEM. *P < 0.05; **P < 0.01; ns no significance. Scale bar, 100 μm.

HEI-OC1 cells with *Fscn2* overexpression (o/e-*Fscn2* cells) were created by infecting the cells with lentiviral particles carrying *Fscn2* gene expression vectors (pSLenti-EF1-EGFP-P2A-Puro-CMV-Fscn2-3xFLAG-WPRE). The increased expression levels of *Fscn2* led to higher cell proliferation and migration rates in o/e-*Fscn2* cells (Fig. 5A–C). Furthermore, Western blot analysis showed that overexpression of *Fscn2* in HEI-OC1 cells resulted in lower level of PARVB and higher levels of p-ILK, p-AKT, and Bcl-2 (Fig. 5D–H).

**Fig. 6:**
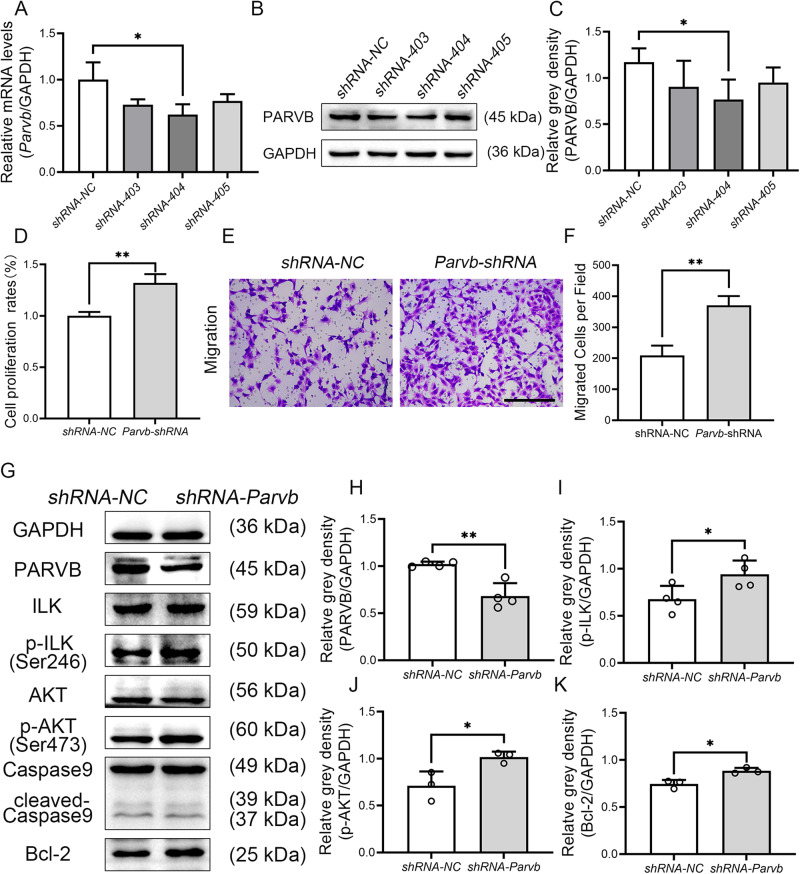
Downregulation of *Parvb* expression in HEI-OC1 cells promoted cell proliferation ability and cell migration rate. **A**–**C** The expression levels of *Parvb* were detected by qRT-PCR and Western blot. **D** Cell viability was assessed by CCK-8 assays. **E**, **F** Migration assay was performed to measure the migration ability of the *Parvb* knockdown cells. Scale bar, 100 μm. **G**–**K** The protein levels were detected in *Parvb* knockdown cells by Western blot. Data are represented as means ± SEM. *P < 0.05; **P < 0.01.

HEI-OC1 cells were infected with lentiviral particles that expressed *Parvb*-shRNA to knockdown the expression of *Parvb* gene. Among the three *Parvb* targeting shRNAs (*Parvb*-shRNA-403, *Parvb*-shRNA-404 and *Parvb*-shRNA-405), *Parvb*-shRNA-404 was chosen for subsequent experiments as it reduced *Parvb* expression most effectively at both RNA and protein levels (Fig. 6A–C). Knockdown of *Parvb* expression resulted in increased cell proliferation and migration rates (Fig. 6D–F). In addition, the levels of p-ILK, p-AKT and Bcl-2 were increased (Fig. 6G–K). These results indicate that FSCN2 inhibits the ILK-AKT-Bcl-2 related pathway by upregulating PARVB expression.

### Activated Rac1/Cdc42 levels were downregulated in HEI-OC1 cells knockdown of *Fscn2* gene

**Fig. 7:**
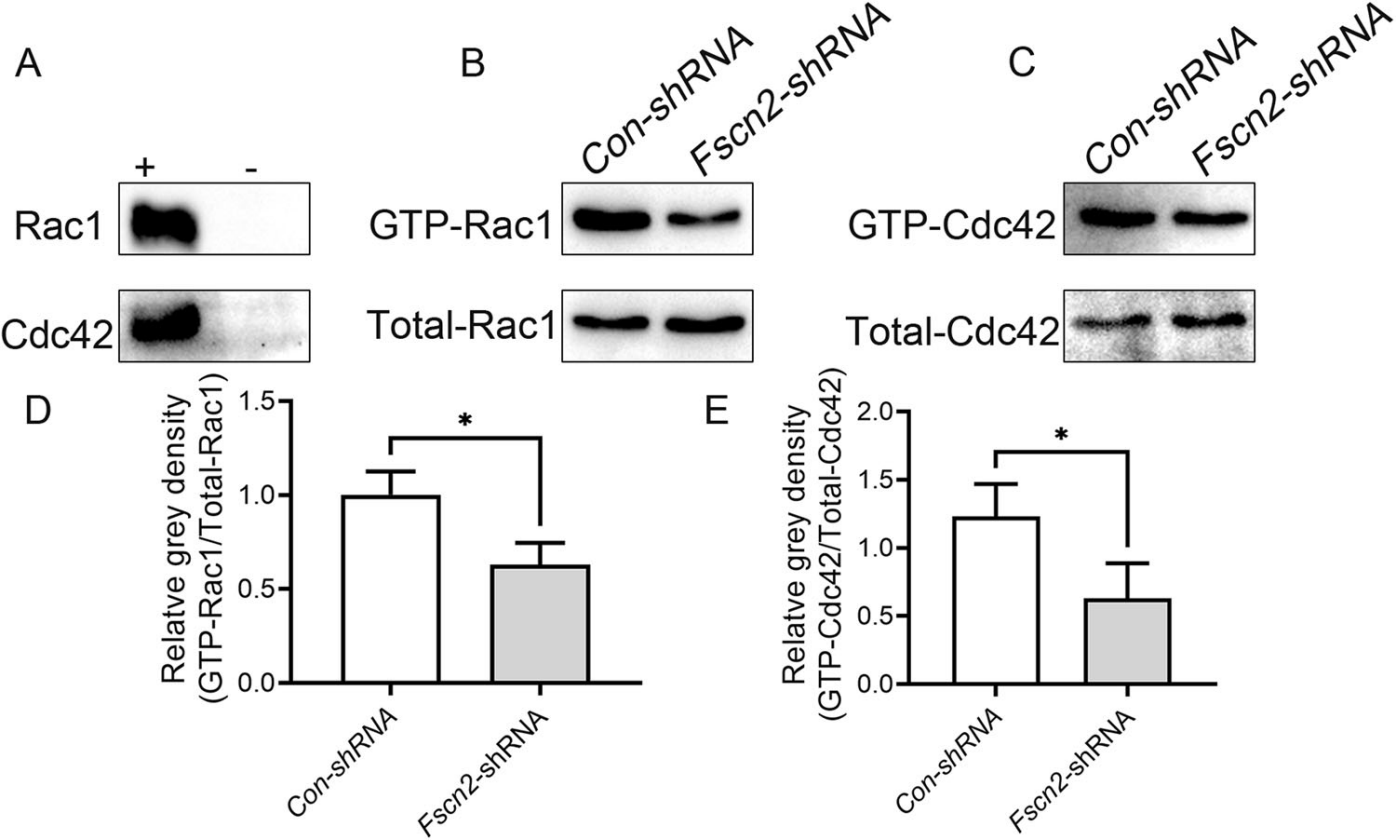
The active levels of Rac1/Cdc42 were reduced in HEI-OC1 cells knockdown of *Fscn2* gene. **A** Negative and positive controls were tested by Western blotting in *Fscn2* knockdown cochlear cells. **B**, **C** Active forms of Rac1 and Cdc42 were detected by pull-down assays in *Fscn2* knockdown cochlear cells. **D** Quantitation of the bands of the active Rac1 on Western blots using the ImageJ software. The bands were normalized to the amount of total Rac1. **E** Quantitation of the bands of active Cdc42 on Western blots using the ImageJ software. The bands were normalized to the amount of total Cdc42. Data are represented as means ± SEM. *P < 0.05.

Among the total Rac1 and Cdc42, the GTP-Rac1 and GTP-Cdc42 possess biological activity, whereas GDP-Rac1 and GDP-Cdc42 can not exert functions. To further explore the effects of FSCN2 on Rac1 and Cdc42 activities, PAK-GST protein beads were used to pull-down GTP-Rac1 and GTP-Cdc42. The results revealed that, by downregulation of *Fscn2* gene in HEI-OC1 cells, the cell migration rate was reduced, and the levels of activated Rac1 and Cdc42 were also downregulated (Fig. 7).

### FSCN2 inhibits PPAR-γ nuclear translocation, thereby regulating PARVB expression in HEI-OC1 cells

**Fig. 8:**
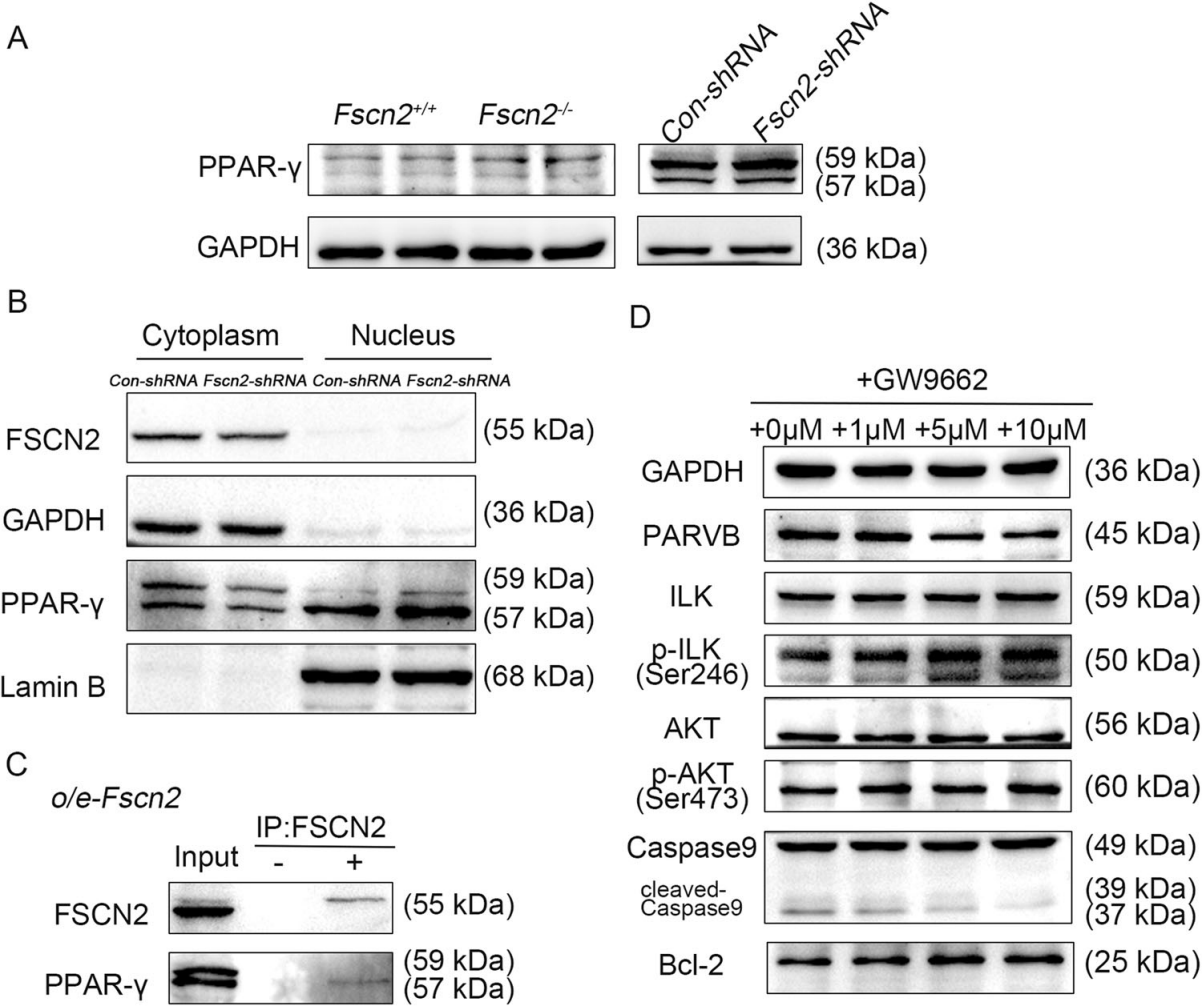
FSCN2 regulates PARVB by inhibiting the translocation of PPAR-γ into the nucleus. **A** PPAR-γ expression in the inner ears of *Fscn2* knockout mice and *Fscn2* knockdown HEI-OC1 cells. **B** The level and localization of PPAR-γ were assessed in *Fscn2* knockdown cells (*Fscn2*-shRNA) by immunoblot assay. GAPDH and Lamin B were used as controls to determine the purity of cytoplasmic and nuclear fractions, respectively. **C** FSCN2-PPAR-γ association was analyzed by immunoprecipitation in o/e-*Fscn2* cells. **D** HEI-OC1 cells were treated with GW9662 (a PPAR-γ inhibitor) for 48 h at concentrations of 1, 5 and 10 μM, respectively. The levels of the indicated molecules were detected by Western blot.

Previous studies have shown that PPAR-γ binds to the peroxisome proliferator response element-like cis-element (ACTAGGACAAAGGACA) in the *Parvb* promoter to promote PARVB expression [26]. To investigate whether FSCN2 regulates PARVB expression by interaction with PPAR-γ, the expression of PPAR-γ in *Fscn2* knockdown cochlear cells was examined. Compared to *Con-shRNA* infected cells, PPAR-γ expression was higher in *Fscn2*-shRNA infected cells (Fig. 8A). Cytoplasmic and nuclear protein isolation tests showed that PPAR-γ was localized in both cytoplasm and nucleus, with a higher level in the nucleus in the *Fscn2*-shRNA infected cells (Fig. 8B). Immunoprecipitation was used to detect the interaction between the two proteins. FSCN2 was found to bind to PPAR-γ in HEI-OC1 cells using anti-flag affinity gels to pull down FSCN2-Flag and its binding proteins (Fig. 8C). Inhibiting PPAR-γ function with GW9662 in HEI-OC1 cells reduced PARVB expression and increased the levels of p-ILK, p-AKT, and Bcl-2 (Fig. 8D). Therefore, FSCN2 may regulate PARVB expression by inhibiting PPAR-γ nuclear translocation, thereby affecting downstream molecule expression and regulating cell survival.

The original Western blots used in the manuscript were provided as Supplemental Material-2 (Figs. S3C; S4D; S5D; S6B, D; S7A–C; S8A–D).

## Discussion

### Upregulation of PARVB is involved in the degeneration of cochlear cells and hearing impairment in *Fscn2*^*−/−*^ mice

Hair cells and spiral neurons in the cochleae are essential for auditory conduction, as they convert sound waves into neural signals and transmit them to the auditory cortex for hearing [27–29]. However, damage to these cells can lead to hearing loss, which can be caused by genetic mutations, noise exposure, ototoxic drugs, inflammation, aging, and other factors [30, 31]. In mammals, damaged cochlear hair cells cannot regenerate, leading to permanent hearing impairment. *Fscn2*^*−/−*^ mouse is a typical model of age-related hearing loss which exhibits progressive hearing impairment and outer hair cell death from 3 weeks of age [16], as well as a progressive degeneration of spiral ganglion neurons from 4 weeks to 40 weeks of age, especially at basal turns. To investigate the molecular mechanism of hearing loss in *Fscn2* knockout mice, gene expression profiling was performed on the inner ears. The results showed that 72 genes were upregulated and 172 genes were downregulated in *Fscn2*^*−/−*^ mice, compared to *Fscn2*^*+/+*^ mice. GO and KEGG analysis of the differentially expressed genes revealed that cell movement, migration and apoptosis regulation signal pathways were the most relevant biological processes. In addition, *Fscn2* was found to be closely related to cell signal transduction pathways such as focal adhesion (PARVB, PAK2, Vav3, Mapk8, Itga4, *ect*.) through KEGG analysis. Further experiments revealed that the expression of PARVB was significantly increased in *Fscn2*^*−/−*^ mice. This is the first demonstration of PARVB expression in the mouse inner ears. We thus predict that upregulation of PARVB contributes to the degeneration of cochlear cells and the hearing impairment in *Fscn2*^*−/−*^ mice. These findings provide valuable insights into the molecular mechanisms underlying hearing loss in *Fscn2*^*−/−*^ mice and may lead to the development of new therapies for hearing impairment.

### Downregulation of p-AKT-related signaling pathway, as a result of upregulation of PARVB, diminishes the survival of cochlear cells

Parvin proteins have no catalytic activity and they function to mediate interacting with a variety of binding proteins, including ILK, a key protein in this pathway [32–34]. PARVB plays a crucial role in integrin-mediated cell adhesion, cell morphology, and cell motility through interaction with the CH2 domain of ILK [35]. ILK forms a heterotrimeric complex with PINCH and Parvins (IPP) that regulates integrin-extracellular matrix interactions and stress fiber formation, as well as coupling structural proteins to signal transduction functions [36]. The ILK, PINCH, and Parvins complex regulates Akt (also known as protein kinase B or PKB), a key component in intracellular signaling pathways [37]. *Parvb* is considered as a tumor suppressor gene, and inhibits breast cancer growth [38]; decreased expression of PARVB in breast cancer cells results in elevated ILK protein and kinase activity levels, suggesting that *Parvb* downregulation stimulates ILK signaling [39]. Immunoprecipitation assays indicates that ILK directly interacts with AKT and promotes cell survival and proliferation by mediating AKT (Ser473) phosphorylation [40]. Knockdown of β-Parvin via small interference RNA (siRNA) increases the photon flux for the interaction between ILK and AKT1, indicating that β-Parvin may be a key regulator of cell survival in the ILK-mediated AKT signaling cascade [41]. While the expression of PARVB in cochlear cells has not been previously reported, our results revealed that PARVB expression was increased in the inner ears of *Fscn2* knockout mice. Immunofluorescence assays demonstrated strong PARVB staining in the spiral ganglion neurons. In the *Fscn2* knockout mice, the levels of p-ILK, p-AKT, and Bcl2 were decreased, while the levels of PARVB and cleaved-Caspase9 was increased in the inner eras. These suggest that increased PARVB level may contribute to cochlear cell death in *Fscn2* knockout mice by inhibiting the ILK-AKT-Bcl2 pathways. This mechanism was further verified in HEI-OC1 cells by knockdown or overexpression of *Fscn2* as well as in cells downregulation of *Parvb*. In addition, knockdown of *Fscn2* reduced cell proliferation ability, whereas overexpression of *Fscn2* or downregulation of *Parvb* increased cells proliferation ability. However, we failed to find a significant increment of cleaved-Caspase 9 or Caspase 3 in the HEI-OC1 cells knockdown of *Fscn2*. Therefore, cochlear cell death in *Fscn2* knockout mice may be caused by increased PARVB expression. Upregulation of PARVB inhibits the phosphorylation of ILK and AKT (Ser473) and downregulates the ILK-AKT-Bcl2 signaling pathway, leading to low cochlear cell survival ability and progressive hearing loss in *Fscn2* knockout mice.

### Downregulation of the ILK-Rac1/Cdc42 signaling inhibits the migration ability of the cochlear cells in vitro

In this study, we observed that the migration ability of HEI-OC1 cells with low level expression of *Fscn2* was decreased, while the migration ability was increased in the cochlear cells overexpressing *Fscn2* or knockdown *of Parvb*, as determined by Transwell assay. Previous studies have shown that PARVB and α-PIX are co-localized to actin structures, such as filopodia and laminar pseudopodia. In addition to interacting with ILK, PARVB can also bind to α-PIX (ARHGEF6) [42]. The PARVB-α-PIX complex activates Rac1/Cdc42, which plays a crucial role in actin reorganization and cell migration [43, 44]. PARVB, ILK and α-PIX can also form a trimeric complex, in which the CH2 domain of PARVB is phosphorylated by ILK, causing a conformational change in the CH1 domain, activating α-PIX and leading to the activation of Rac1/Cdc42 and actin cytoskeletal remodeling. This process is ILK activity dependent, as inactive ILK cannot form the trimer and activate Rac1/Cdc42 [45]. Based on these findings, we hypothesized that the decreased activity of ILK could lead to decreased levels of activated Rac1/Cdc42, which were responsible for the reduction of migration ability of HEI-OC1 cells knockdown of *Fscn2*. To test this, we examined the levels of Rac1/Cdc42 in the cochlear cells knockdown of *Fscn2* and found that activated Rac1/Cdc42 was indeed decreased. Therefore, we propose that downregulation of ILK-Rac1/Cdc42 signaling by reduced FSCN2 expression contributes to the decreased migration ability of cochlear cells in vitro. The relation of reduced HEI-OC1 cell migration ability in vitro and cochlear cell loss in *Fscn2* knockout mice needs further investigation.

### FSCN2 regulates the expression of PARVB by inhibiting the nuclear translocation of PPAR

Peroxisome proliferator activated receptors (PPARs) are a class of transcription factors, with PPAR-γ being an intracellular receptor that is regulated by endogenous and exogenous repressors and activators. PPAR-γ forms a heterodimer with the retinoic acid X receptor (RXR), by binding to its ligands, which activates transcription of specific genes in the nucleus [46, 47]. Studies have shown that PPAR-γ regulates *Parvb* expression by binding to a peroxisome proliferator response element (PPRE)-like cis-element in the *Parvb* promoter [26]. In the present study, we observed that the expression of PARVB was increased in the inner ears of *Fscn2* knockout mice. Further investigation revealed that the expression levels of PPAR-γ were also increased in the inner ears of *Fscn2* knockout mice and in *Fscn2* knockdown HEI-OC1 cells. Treatment of HEI-OC1 cells with a PPAR-γ inhibitor reduced the expression levels of PARVB, suggesting that FSCN2 downregulates the expression of PARVB by affecting the action of PPAR-γ. In addition, immunoprecipitation experiments showed that FSCN2 could bind to PPAR-γ, and nuclear cytoplasmic separation assays indicated that the amount of PPAR-γ in the nucleus was increased in *Fscn2* knockdown cells. We thus propose that FSCN2 regulates the level of PARVB by inhibiting PPAR-γ dependent *Parvb* transcription. FSCN2 contains four tandem cloverleaf folding domains which may allow it to bind to PPAR-γ through protein interactions and act as a repressor to inhibit PPAR-γ from binding to its ligands or entering the nucleus as a heterodimer. Our findings demonstrate that FSCN2 is not only a structural protein but also a regulatory protein that modulates PPAR-γ to control the expression of PARVB.

## Conclusion

Our study has demonstrated that the loss of FSCN2 function results in an increment in PARVB expression in the mouse inner ears. Upregulation of PARVB contributes to the cochlear cell death and hearing loss in the *Fscn2* knockout mice by downregulating ILK-AKT related signaling pathways. FSCN2 regulates PARVB expression by inhibiting the nuclear translocation of PPAR-γ. This study may provide new targets and ideas for the prevention and treatment of genetic hearing loss.

## Materials and methods

### Mice

The wild-type (*Fscn2*^*+/+*^) mice and *Fscn2*^*−/−*^ mice were used in this study. PCR genotyping was performed using the primers shown in Table 1. The study was approved by the Animal Use and Care Committee of Binzhou Medical University. A total of 213 littermates aged 4–40 weeks were included in the study, with 112 *Fscn2*^*−/−*^ mice in the experimental groups and 101 wild-type mice in the control groups. The animals were randomly distributed over different experimental conditions and blinding was applied during data acquisition and analysis.

**Table 1:** PCR primers for mouse genotyping.

Primer ID	Primer sequences
Fscn2-KF	5′-ATTGGAGCAGGTAGCGTCCATGTC-3′
Fscn2-KR	5′-TCACAGGCCACACGTCCATCTTC-3′

### Hematoxylin and eosin (HE) staining

After intraperitoneal injection of 2% tribromoethanol (0.2 mL/10 g of body weight) for anesthesia, the inner ears of mice were removed and fixed in 4% paraformaldehyde at 4 °C for 24 h. Decalcification was performed using 10% EDTA for 3–7 days. Tissues were dehydrated, embedded in paraffin, and cut into 5 µm sections. Hematoxylin and eosin (HE) staining was performed on the sections, and spiral ganglion neurons (SGNs) were counted in the apical, middle, and basal regions of the cochlear sections using a 40 × objective light microscope (BX53, Olympus, Japan). The density of SGNs was presented as the number of cells per square millimeter. Counts of SGNs were carried out following the methods described previously [48].

### Microarray analysis

*Fscn2*^*+/+*^ mice (*n* = 5) and *Fscn2*^*−/−*^ mice (*n* = 6) at 8 weeks of age were randomly selected. After genotyping and detection of the ABR thresholds, total RNA was extracted from the inner ears of each of the 11 mice and purified using the QIAGEN RNeasy® Kit. The concentration and purity of RNA were detected by NanoDrop ND-2000 (Thermo Fisher Scientific, USA), and the integrity of RNA was detected by Agilent Bioanalyzer 2100 (Agilent Technologies Co., LTD.). Three qualified samples from each group (Table 2) were selected for subsequent microarray hybridization. The total RNA of the inner ears was reverse-transcribed to form double-stranded cDNA, and the synthesized cRNA was labeled with Cyanine-3-CTP (Cy3), which were then fragmented and subjected to microarray hybridization. The original images were obtained by Agilent Scanner G2505C (Agilent Technologies Co., LTD.). Feature Extraction software was used to complete the original image processing, and then the original data of chip hybridization were extracted. The original data were processed simultaneously by using Genespring software. Differential genes were screened by the *P* value and Fold change (FC) value of *t*-test, and the screening criteria were set as FC value ≥ 2.0 and *P* value ≤ 0.05 for those with increased or decreased FC value. According to the results of microarray analysis, the differentially expressed genes were screened, and the primers for reverse transcription polymerase chain reaction (RT-PCR) were designed for verification.

**Table 2:** Qualified samples from each group for microarray analysis.

Mouse lines	Sample name	Density	A260/ 280	A260/230	Volume	Quantity	2100		Results
		(μg/μl)			(μl)	(μg)	28 S/18 S	RIN	
*Fscn2*^*+/+*^	02	0.2344	2.14	1.85	20	4.69	1.8	8.7	A
	03	0.2177	2.13	1.87	20	4.35	3.0	8.1	A
	04	0.2687	2.14	1.87	20	5.37	1.5	8.0	A
*Fscn2*^*−/−*^	06	0.3186	2.15	1.80	20	6.37	2.0	8.9	A
	07	0.2853	2.14	1.87	20	5.71	2.5	9.2	A
	08	0.2878	2.14	1.80	20	5.76	2.1	8.7	A

### Real-time RT-PCR

Total RNA was extracted from cells or tissues using TRIzol reagent (Invitrogen; Thermo Fisher Scientific, USA). Complementary DNA (cDNA) was synthesized using the Transcriptor First Strand cDNA Synthesis Kit (Roche, 04896866001, Switzerland). cDNA was used for quantitative PCR (qPCR) using a SYBR Green PCR Master Mix Reagent Kit (Roche, Basel, Switzerland) in the MyiQTM Real-Time PCR Detection System (Bio-Rad Laboratories, Inc., Hercules, CA). The RT reactions were performed as follows: 25 °C for 10 min, 55 °C for 30 min, and 85 °C for 5 min. PCR amplification was performed in a thermal cycler for 40 cycles at the following cycle conditions: 95 °C for 10 s, 60 °C for 30 s, and 72 °C for 30 s. The expression of *Gapdh* was detected as the endogenous control. Primer sequences are shown in Table 3.

**Table 3:** Primers of real-time RT-PCR for *Parvb* gene.

Primer ID	Primer sequences
Parvb-F	5′-CCCAAGATGAAGAAGGACGA-3′
Parvb-R	5′-AGGGTGAATGTCCACCAAAG-3′
Gapdh-F	5′-CTTCCGTGTTCCTACCCCCAATGT-3′
Gapdh-R	5′-GCCTGCTTCACCACCTTCTTGATG-3′

### Western blotting

Whole-cell lysates were prepared in RIPA lysis buffer containing 1 mM phenylmethylsulfonyl fluoride (PMSF, Solarbio, R0010, China). Detection of phosphorylated proteins requires the addition of phosphorylase inhibitors (Roche, 04906845001, Switzerland). Protein concentrations were determined using a BCA Protein Assay Kit (Solarbio, PC0020, China). Proteins (30 μg) were electrophoresed on 10–12% SDS-PAGE gels and were transferred to PVDF membranes, which were blocked in 5% BSA or skim milk for 2 h and were incubated with primary antibodies at 4 °C overnight. The membranes were then incubated with secondary antibodies at room temperature for 2 h. Next, the membranes were washed three times with 1×TBST solution. The reaction signals were detected using an ECL detection system (Novland, NCL5079, China). The following antibodies were used in this study: anti-Fascin2 (Abcam, ab232768), anti-PARVB (Proteintech, 14463-1-AP), anti-PPAR gamma (Abcam, ab45036), anti-beta-Parvin (Santa Cruz Biotechnology, sc-374581), anti-AKT (Proteintech, 10176-2-AP), anti-p-AKT (Cell Signaling Technology, #4060 S), anti-ILK (Santa Cruz Biotechnology, sc-20019), anti-ILK (Proteintech, 12955-1-AP), anti-p-ILK (Bioss, bs-5444R), anti-Caspase9 (Cell Signaling Technology, #9504), anti-Bcl-2 (Proteintech, 26593-1-AP), anti-GAPDH (Proteintech, 10494-1-AP), anti-beta Actin (Abcam, ab8227), anti-Lamin B (Wanleibio, WL01775, China), goat anti-rabbit IgG H&L(HRP) (Abcam, ab97051), and goat anti-mouse IgG-HRP (Absin, abs20001).

### Immunohistochemical staining

The inner ear tissues were fixed, dehydrated, embedded in paraffin, and sectioned (5 μm). The tissue slides were dewaxed in a graded alcohol and xylene. Antigen retrieval was performed by heating in citrate buffer and endogenous peroxidase activity was blocked by 3% H_2_O_2_. Tissue slides were permeabilized using 0.5% Triton X-100 for 30 min and blocked with 5% goat serum in PBS for 1 h. The tissue slides were then incubated with an antibody against p-AKT (CST, 4060 s, 1:100) at 4 °C overnight. Slides were then incubated with biotinylated goat anti-rabbit secondary antibody at room temperature for 30 min, followed by 30 min incubation with biotin-streptavidin. The peroxidase reaction was performed by incubation in DAB solution at room temperature for 2 min. Finally, all sections were stained with hematoxylin for 30 s and rehydrated in a graded alcohol. The stained tissues were imaged using a 40× objective under the microscope.

### Immunofluorescence staining

After fixation and decalcification, the inner ears were embedded in OCT. The OCT-embedded cochlear specimens were sliced into 6 µm sections using a freezing microtome (Leica, CM1950, Germany) at −20 °C and mounted on silane-coated slides. The tissue slides were fixed in 4% paraformaldehyde for 20 min, washed three times for 5 min each with PBS, and blocked with 5% goat serum in PBS for 1 h. The tissue slides were then incubated with an antibody against PARVB (Santa Cruz Biotechnology, sc-374581, 1:100) overnight at 4 °C. On the next day, the tissue slides were washed three times for 5 min each with PBS and incubated with a secondary antibody (Abcam, ab150113, 1:400) at room temperature for 2 h. The tissue slides were washed three times in PBS and counterstained with DAPI. The stained tissues were imaged using a 40× objective under a confocal microscope (LSM880, Zeiss, Germany).

### Cell culture

Mouse auditory cell lines, HEI-OC1 (House Ear Institute-organ of Corti 1), were kindly provided by F. Kalinec at the House Ear Institute (Los Angeles, CA, USA) and Qingyin Zheng at Case Western Reserve University. The HEI-OC1 cells were cultured under permissive conditions (33 °C, 10% CO_2_) in DMEM (Pricella, PM150210, China) containing 10% FBS (Pricella, 164210, China) without antibiotics. HEI-OC1 cells knockdown of *Fscn2* or overexpression of *Fscn2* were constructed using previously described methods.

### Infection of HEI-OC1 cells with *Parvb*-shRNA-lentivirus

Recombinant plasmids, pLV3-H1-GFP-TA2-puro-shRNA-*Parvb* or pLV3-H1-GFP-TA2-puro-shRNA-NC, were constructed (Shanghai GenePharma, China) to express short hairpin RNA (shRNA) targeting the mouse *Parvb* gene or a negative control (NC) sequence. The DNA sequences for shRNA are listed in Table 4. HEI-OC1 cells were seeded in six-well plates, followed by overnight incubation at 37 °C. The virus were diluted with DMEM medium at 1:10 and then added in the plates. Puromycin was added to a final concentration of 5 μg/ml. 24 h later, the virus diluent was removed and replaced with 2 ml fresh medium. The cells were cultured at 33 °C in the culture dish (6 cm) for 72 h, and the stable cell lines were obtained after screening with 5 μg/ml of puromycin for 1 week. RNA and protein were extracted to identify the *Parvb*-shRNA-lentivirus infected cells with the highest efficiency.

**Table 4:** DNA sequences of shRNA for targeting mouse *Parvb* gene.

shRNA ID	DNA sequences for shRNA
NC	5′-GTTCTCCGAACGTGTCACGT-3′
Parvb-293	5′-GGAGTTGGTCAAGGTACTTCT-3′
Parvb-559	5′-GGAAGAACCTAGTGGCCATTC-3′
Parvb-1042	5′-GCCCTGAAGATGTGGTGAACT-3′

### Cell viability assay by CCK-8

Cell viability was assessed using a CCK-8 assay kit (Biosharp, BS350B, China). HEI-OC1 cells were counted using an automated cell counter (Invitrogen Countess, Bothell, WA, USA), and adjusted to a concentration of 1.0 × 10^4^ cells/ml. The cells were seeded into a 96-well cell culture plate (100 μl per well) and incubated overnight for attachment. After incubation for 24 h, 10% CCK-8 was added to the wells and incubated for 2 h. The absorbance at 450 nm was measured using a microplate reader (INFINITE 200 PRO, Tecan, Switzerland). The average optical density (OD) value in the control cells was used as the baseline for 100% viability.

### Migration assays

Migration assays were performed using transparent polyethylene membrane cell culture inserts with an 8.0 µm pore size (Corning, CLS3464, USA) following the manufacturer’s instructions. HEI-OC1 cells (4 × 10^3^) were seeded in 100 μl serum-free medium per well in the upper chamber, and medium containing 10% fetal bovine serum was added to the lower chamber (750 μl). After incubation at 33 °C for 24 h, the cells were fixed with 4% paraformaldehyde (Biosharp, BL539A, China) and stained with 0.1% crystal violet solution (Solarbio, C8470, China). Stained cells that migrated through the membrane were counted in five random areas under a microscope using a 20× objective. The cells at five randomly chosen areas were calculated for migration assays.

### Drug treatment

HEI-OC1 cells were seeded into a culture dish (6 cm, 8 × 10^5^ per dish) and incubated overnight for attachment. GW9662 (MedChemExpress, HY-16578, USA), an antagonist of PPAR-γ [49], was added to the cell culture dishes at concentrations of 1 μM, 5 μM, and 10 μM, respectively. 48 h later, the cells grown to 80–90% confluence were lysed on ice with RIPA buffer containing 1 mM PMSF for 30 min. After being centrifuged at 14,000 × *g* for 10 min, the supernatant was collected and stored at −20 °C.

### Immunoprecipitation

The experiments were carried out following the method described [50]. HEI-OC1 cells overexpressing *Fscn2* were cultured to 90% confluence in a 10 cm culture dish, and lysed on ice with 1 ml RIPA buffer and PMSF for 30 min. After centrifugation, the supernatant was collected and temporarily placed in a refrigerator at 4 °C. Anti-DYKDDDDK (Flag) Affinity Gel was used following the manufacturer’s instructions. 1 ml of the cell lysate was added in the Gel overnight. Proteins bound to the gel were eluted off using acid elution the next day. The proteins were denatured by boiling with 2× buffer for 5 min, followed by Western blotting.

### Nuclear and cytoplasmic protein extraction

HEI-OCI cells knockdown of *Fscn2* were cultured to 80% confluence in a 10 cm culture dish. Nuclear and cytoplasmic protein was extracted by using the Nuclear and Cytoplasmic Protein Extraction Kit (Beyotime, P0027, China) following the manufacturer’s protocol. 100 μl of cytoplasmic protein extraction Reagent A supplemented with PMSF was added for cell precipitation on ice for 20 min. Then 10 μl of cytoplasmic protein extraction reagent B was added to the cell precipitation. The supernatant obtained after centrifugation (14,000 × *g*, 10 min) contains the cytoplasmic proteins. The precipitate obtained after centrifugation was added to 50 μl of nuclear protein extraction reagent supplemented with PMSF. After incubation on ice for 30 min, the nuclear proteins were obtained by centrifugation at 14,000 × *g* for 10 min.

### Assay for Rac1/Cdc42-GTPase activity

Rac1 or Cdc42 activities were evaluated using a Rac1/Cdc42 activation assay combo kit from Cell Biolabs (Cell BioLabs, Sand Diego, CA, USA). This process was carried out in accordance with the manufacturer’s protocol and described previously [51]. HEI-OC1 cells were lysed in 1 mL of 1× assay/lysis buffer supplied with the kit. Equal concentrations of samples from each of the cell lysates were used to determine the total amounts of Rac1 and Cdc42 by Western blot. Pull down assays were performed separately to determine the Rac1 and Cdc42 activities using 500–1000 μg of protein from the remaining cell lysates. GTPγS or GDP (supplied with the assay kit) was mixed with the cell lysates and used as either a positive or negative control, respectively. The active and total Rac1 and Cdc42 levels were detected by Western blot using anti-Rac1 or anti-Cdc42 mouse antibodies supplied with the kits.

### Statistical analysis

Data were analyzed using GraphPad Prism 9.0 software (San Diego, CA, USA). *P* < 0.05 was defined as statistically significant, compared with the control or indicated group. A two-tailed Student’s *t*-test (for two groups), one-way ANOVA (for three or more groups) and two-way ANOVA (for two or more groups with two variables) were used for statistical analysis. All data were generated from three or more independent experiments.

### Supplementary information

Supplemental Material-1

Supplemental Material-2

## Data Availability

The data that used and analyzed in this study are available from the corresponding authors upon reasonable request.
