# Supplementary material for: Inhibition of the ILK-AKT pathway by upregulation of PARVB contributes to the cochlear cell death in Fascin2 gene knockout mice
Source: Cell Death Discov. 2024 Feb 19;10:89. doi: 10.1038/s41420-024-01851-5 (PMC10876960; doi:10.1038/s41420-024-01851-5)
Supplement: Supplementary file 1 — Supplemental Material-1 [file 41420_2024_1851_MOESM1_ESM.pdf]

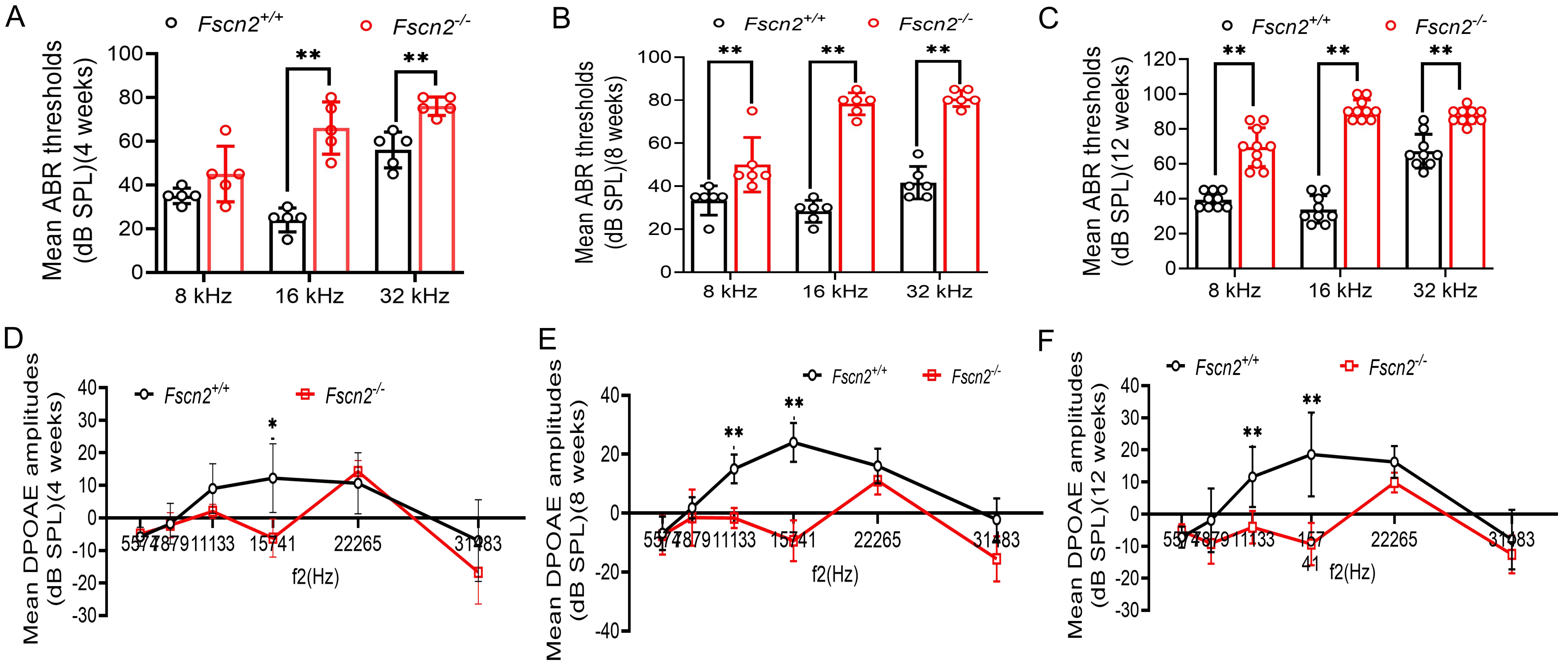


**Fig. S1.** Detection of the mouse ABR threshholds and DPOAE amplitudes at 4, 8 and 12 weeks of age. **A-C** ABR thresholds of *Fscn2*$^{-/-}$ and *Fscn2*$^{+/+}$ mice under different audio stimuli at 4, 8 and 12 weeks of age. **D-F**. DPOAE amplitudes of *Fscn2*$^{-/-}$ and *Fscn2*$^{+/+}$ mice at 4, 8 and 12 weeks of age. **P* < 0.05, ***P* < 0.01

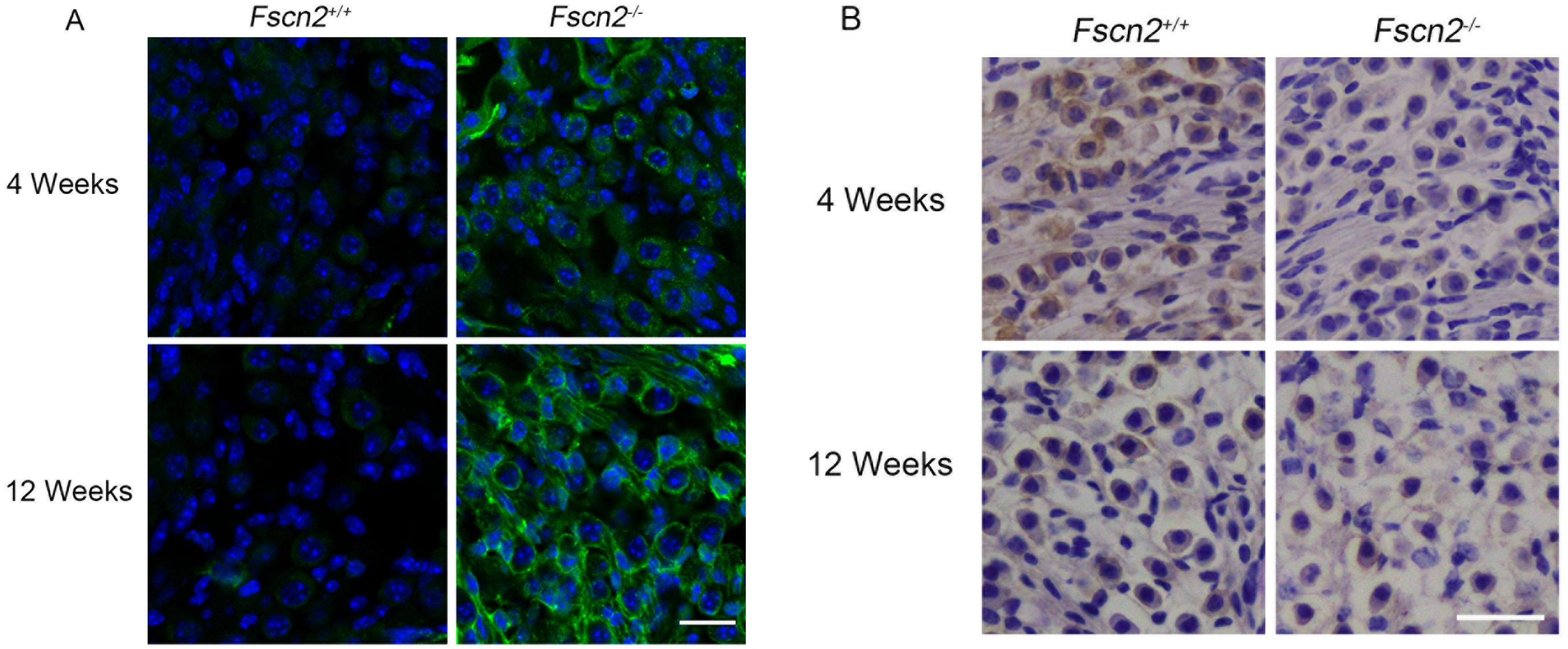


**Fig. S2.** Expression of PARVB and p-AKT in the mouse inner ears of *Fscn2*$^{+/+}$ and *Fscn2*$^{-/-}$ mice at age of 4 and 12 weeks. **A** The expression of PARVB in the spiral ganglion neurons (SGNs) in the cochleae of *Fscn2*$^{+/+}$ and *Fscn2*$^{-/-}$ mice was detected by immunofluorescence histochemical analysis. Scale bar, 20 μm. **B** The expression of p-AKT was detected by immunohistochemical staining in the cochleae of *Fscn2*$^{+/+}$ and *Fscn2*$^{-/-}$ mice. Scale bar, 50 μm

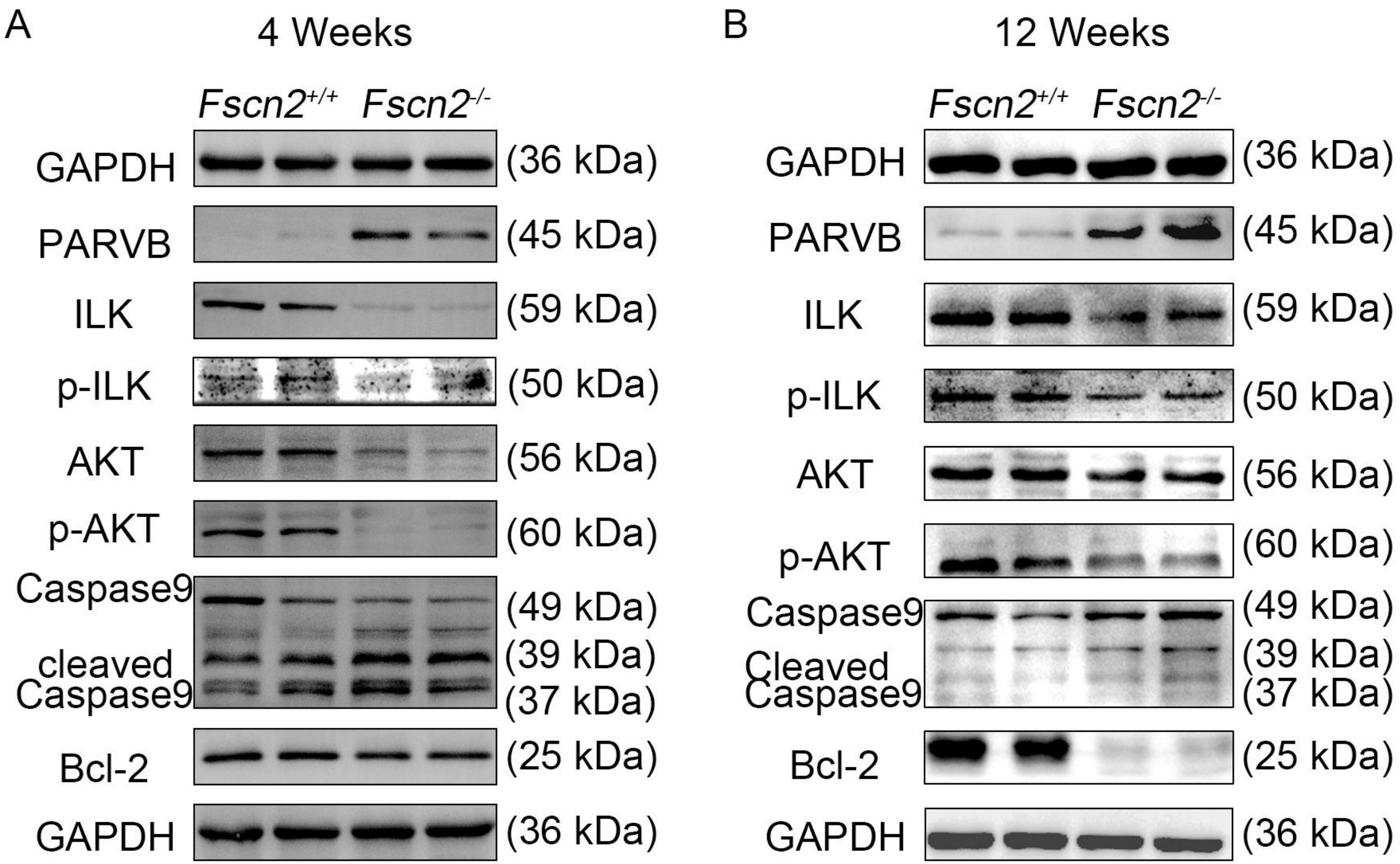


**Fig. S3.** Detection of PARVB associated proteins in the inner ears of *Fscn2*$^{+/+}$ and *Fscn2*$^{-/-}$ mice at age of 4 and 12 weeks. **A** The levels of PARVB, ILK, p-ILK, AKT, p-AKT, cleaved Caspase9 and Bcl-2 were detected by Western blot in the inner ears of *Fscn2*$^{-/-}$ and *Fscn2*$^{+/+}$ mice at 4 weeks of age. **B** The levels of PARVB, ILK, p-ILK, AKT, p-AKT, cleaved Caspase9 and Bcl-2 were detected by Western blot in the inner ears of *Fscn2*$^{-/-}$ and *Fscn2*$^{+/+}$ mice at 12 weeks of age.
