# Supplementary figures and images for: Inhibition of the ILK-AKT pathway by upregulation of PARVB contributes to the cochlear cell death in Fascin2 gene knockout mice
Source: Cell Death Discov. 2024 Feb 19;10:89. doi: 10.1038/s41420-024-01851-5 (PMC10876960; doi:10.1038/s41420-024-01851-5)

Fig. S3C

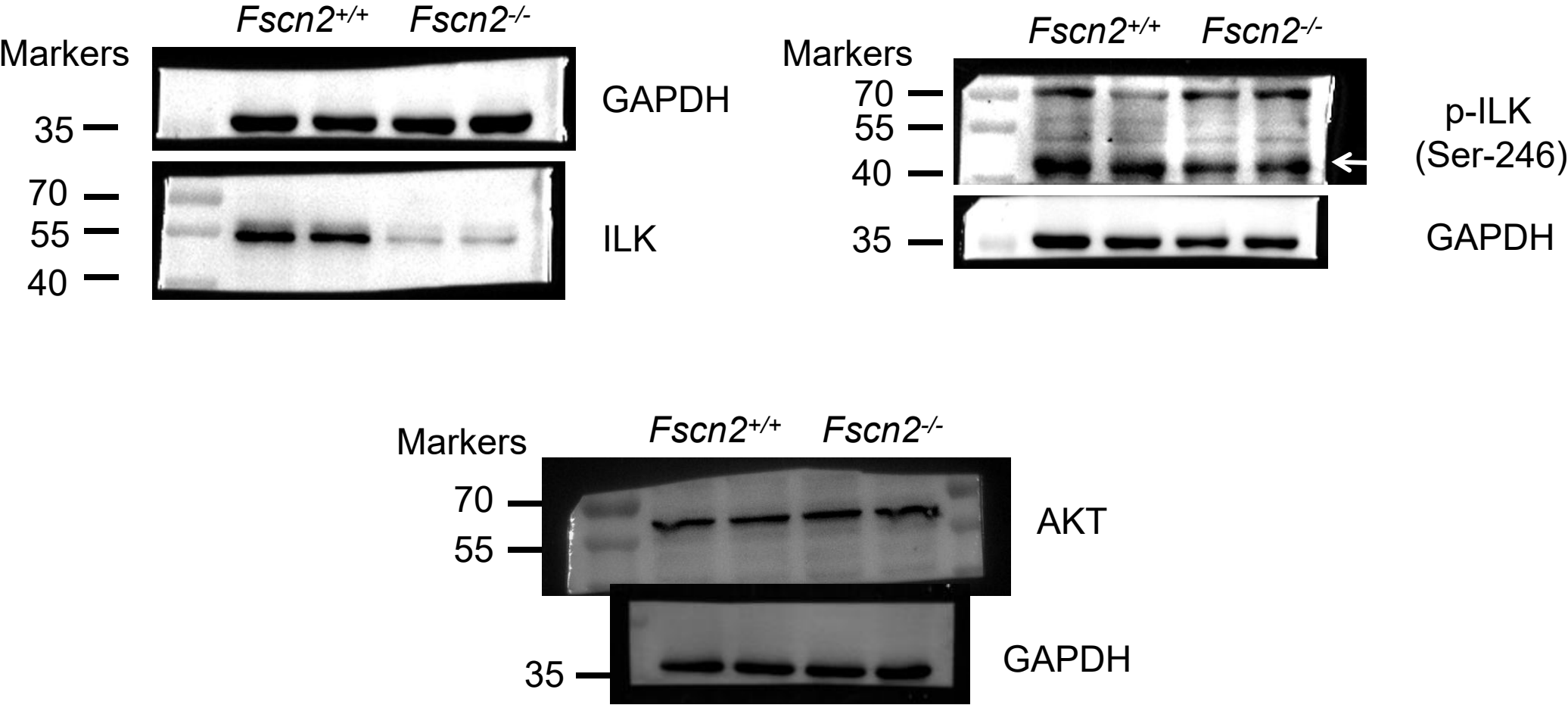

Fig. S3C

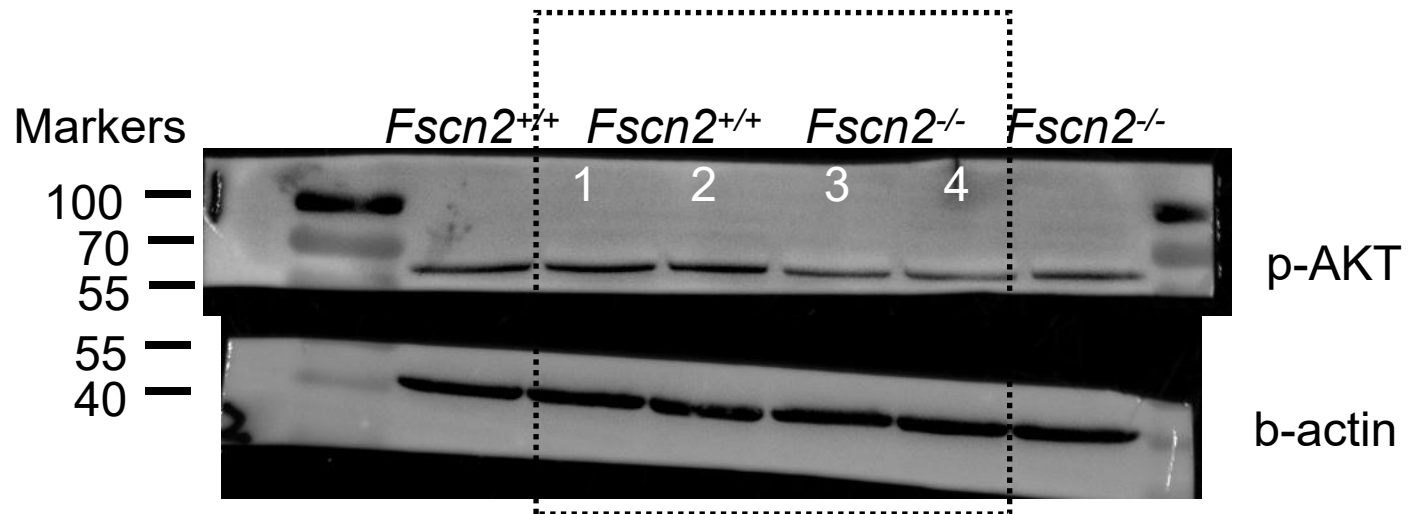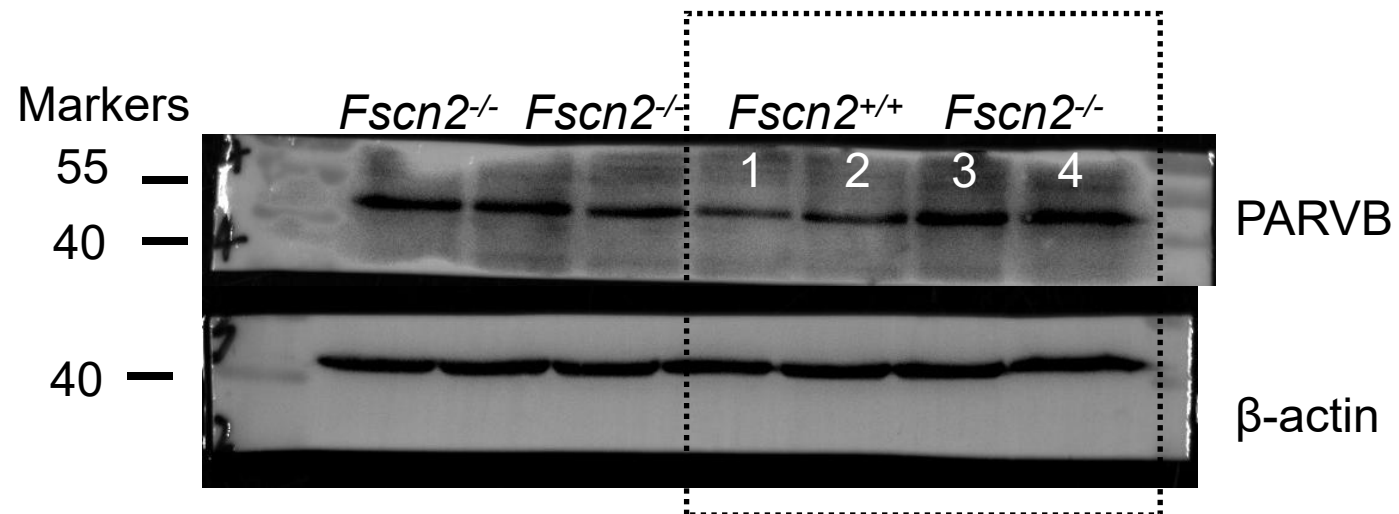

Fig. S3C

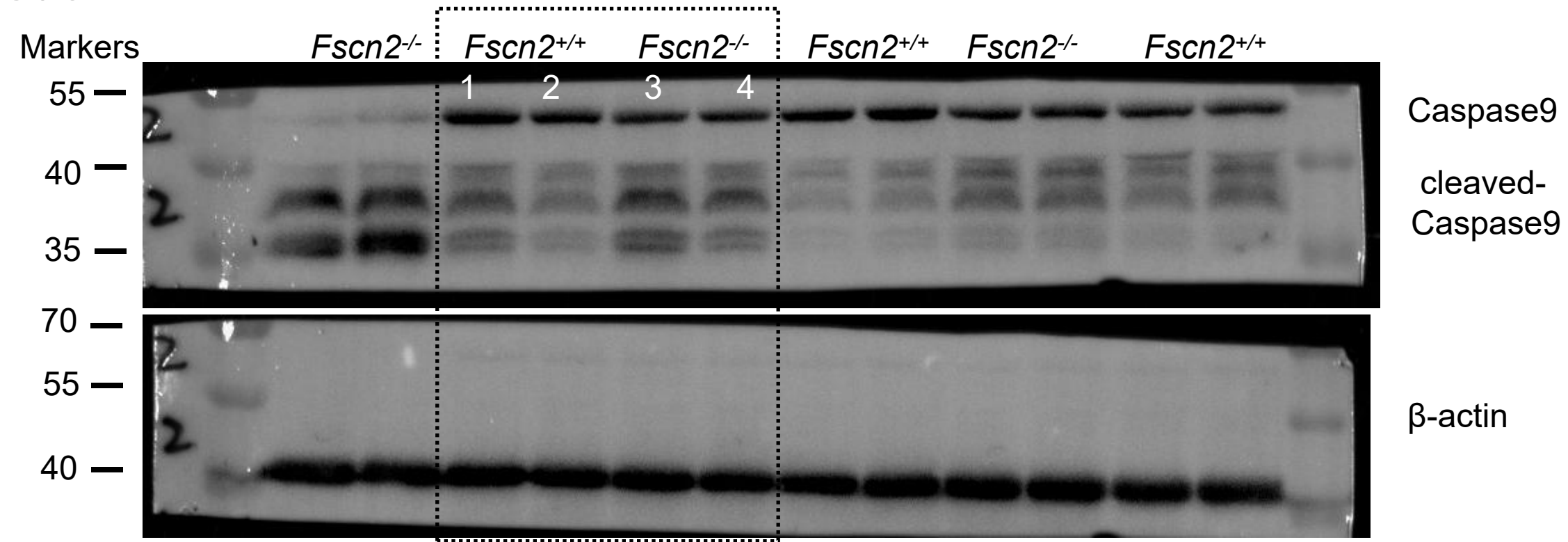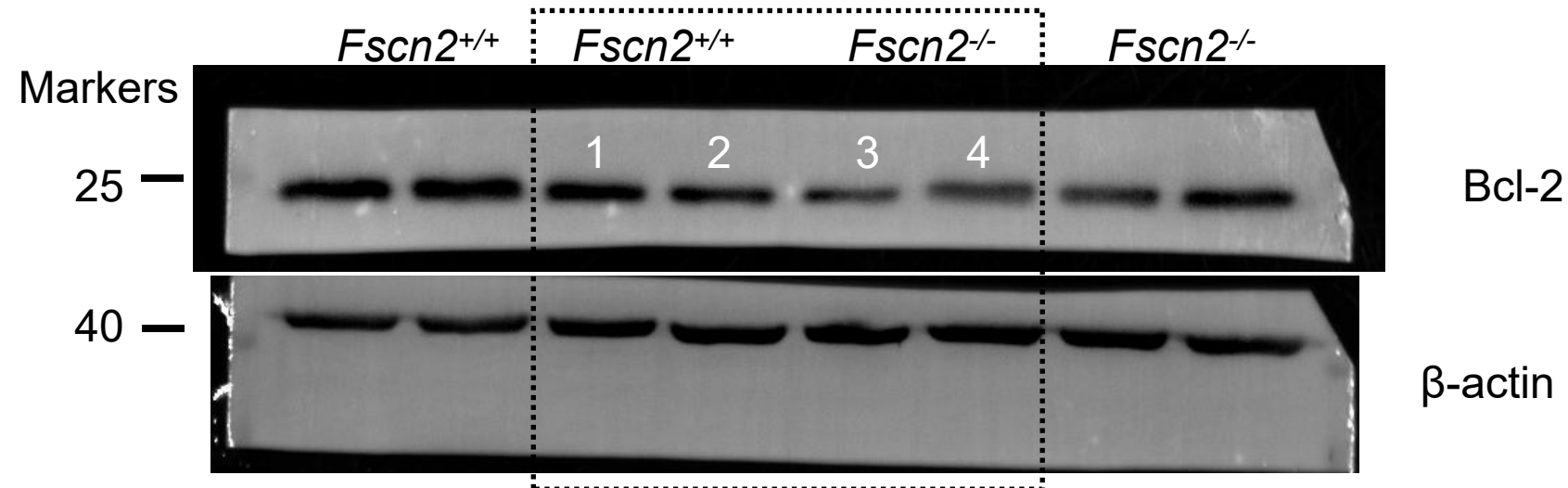

Fig. S4D

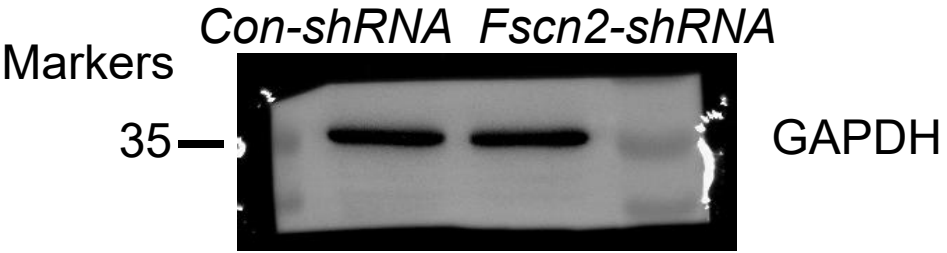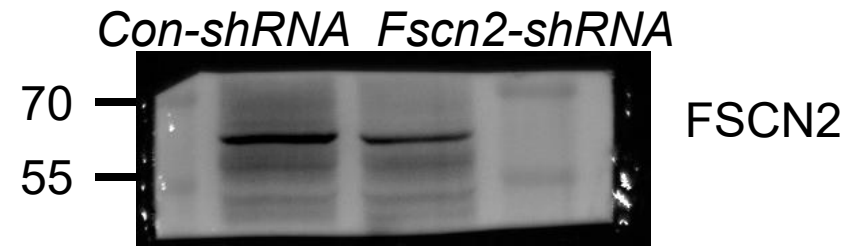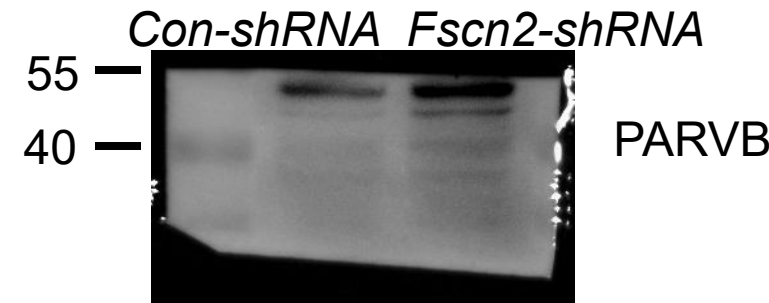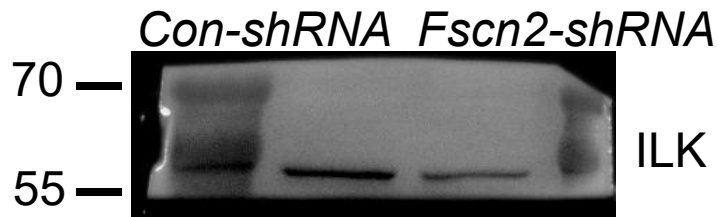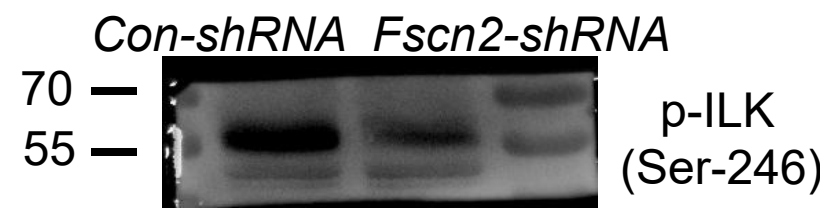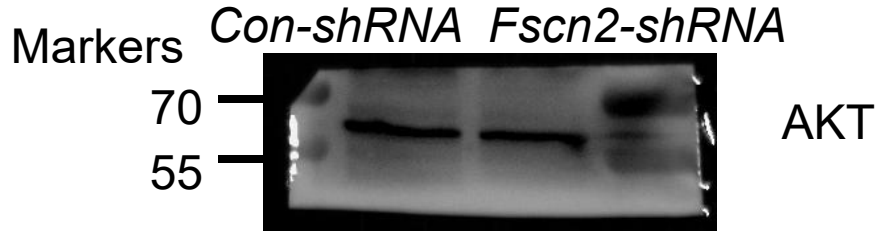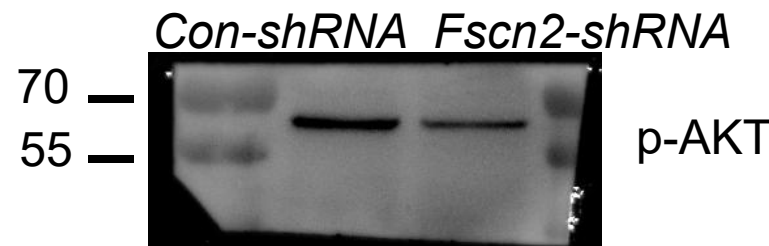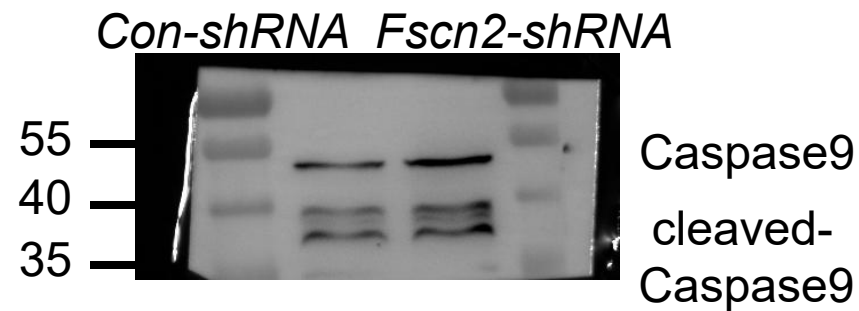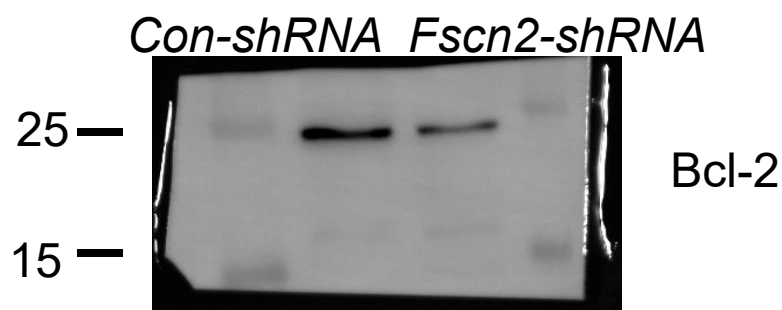

Fig. S5D

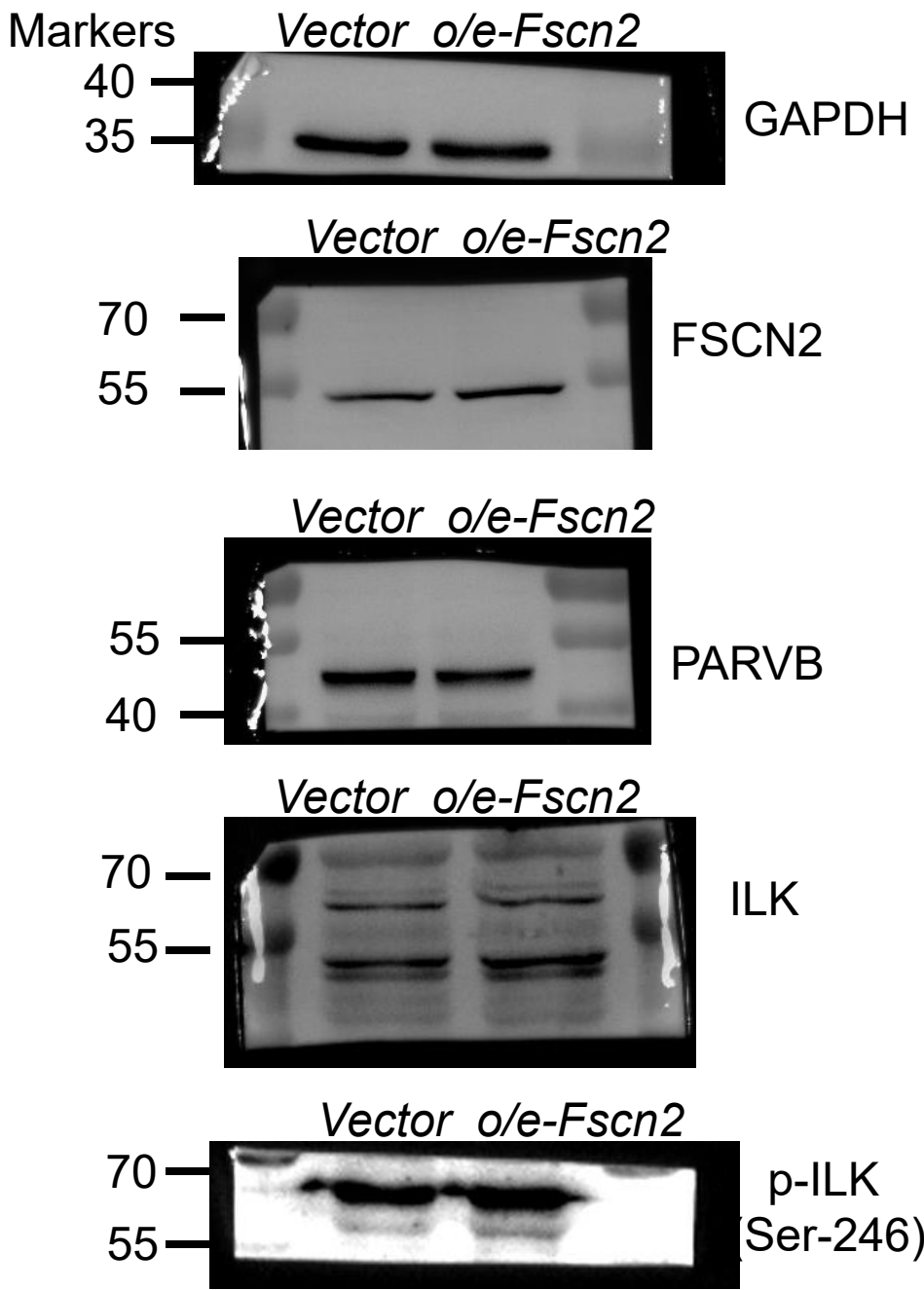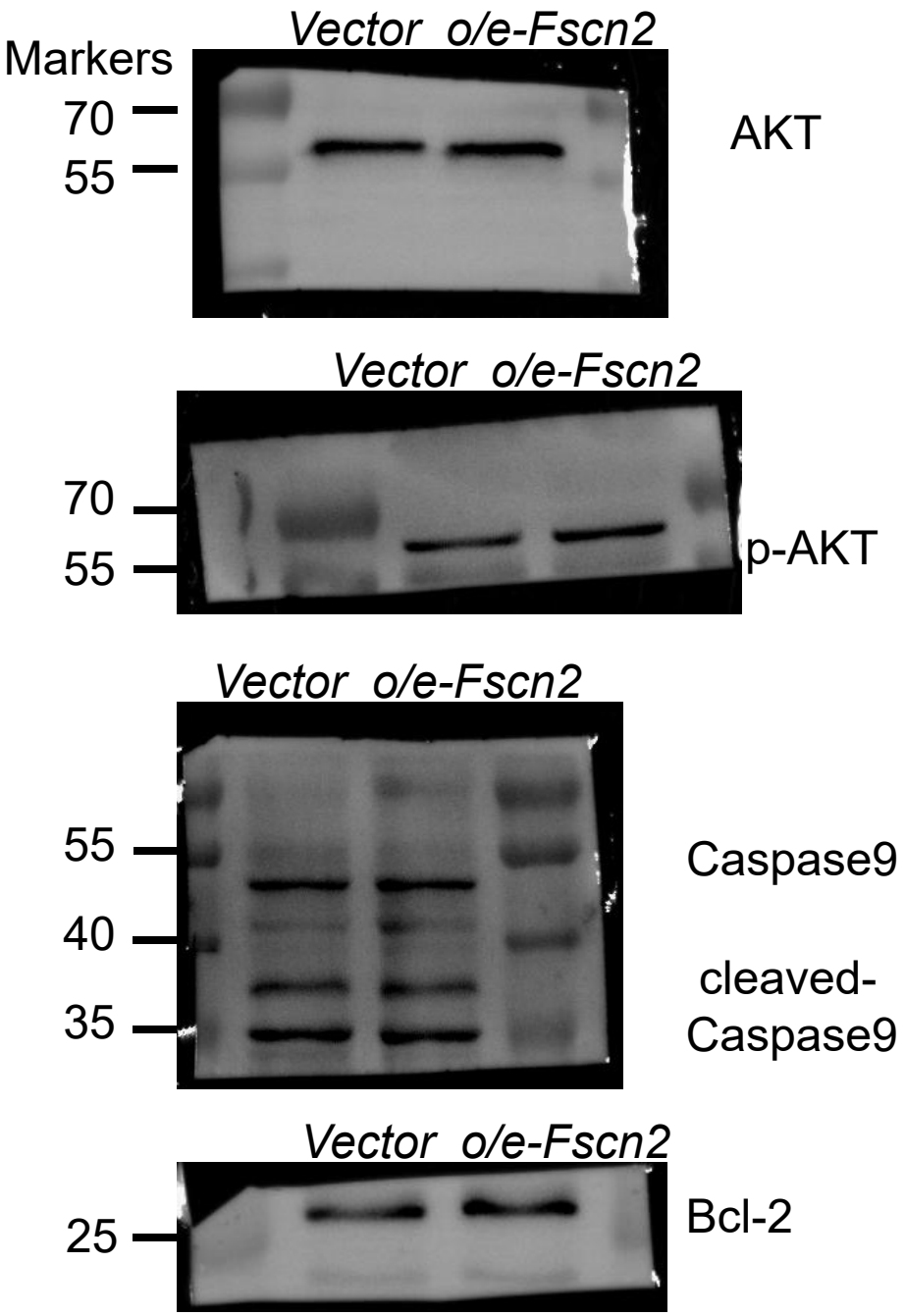

Fig. S6B

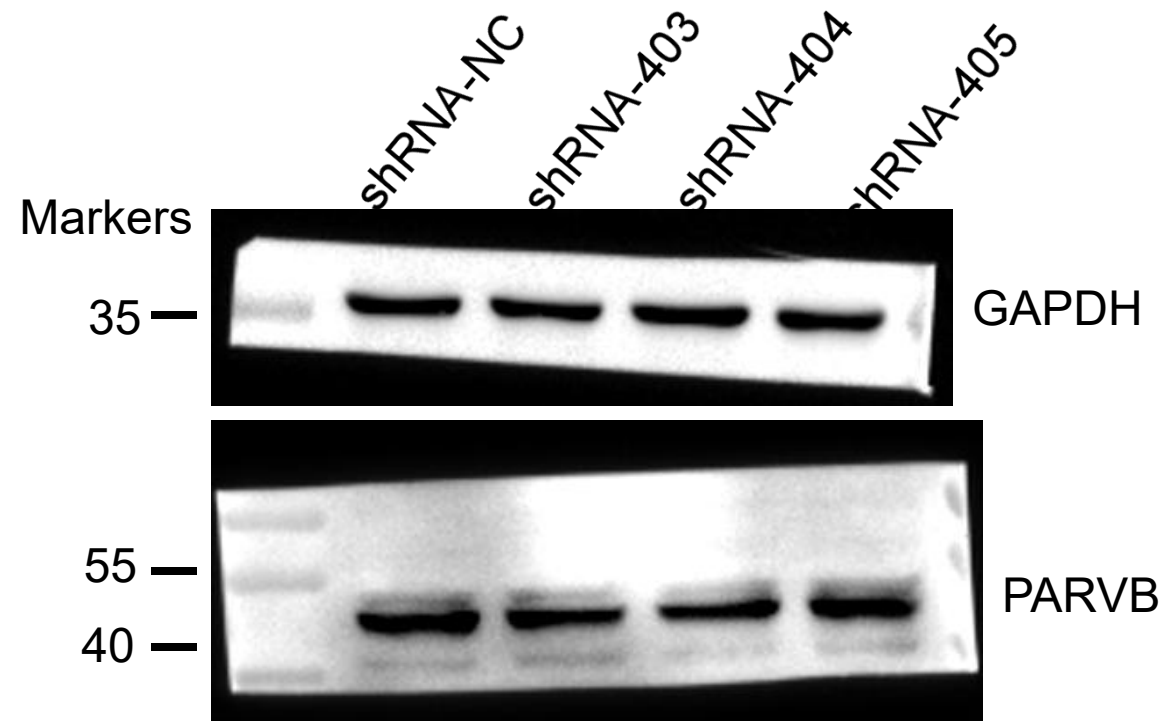

Fig. S6D

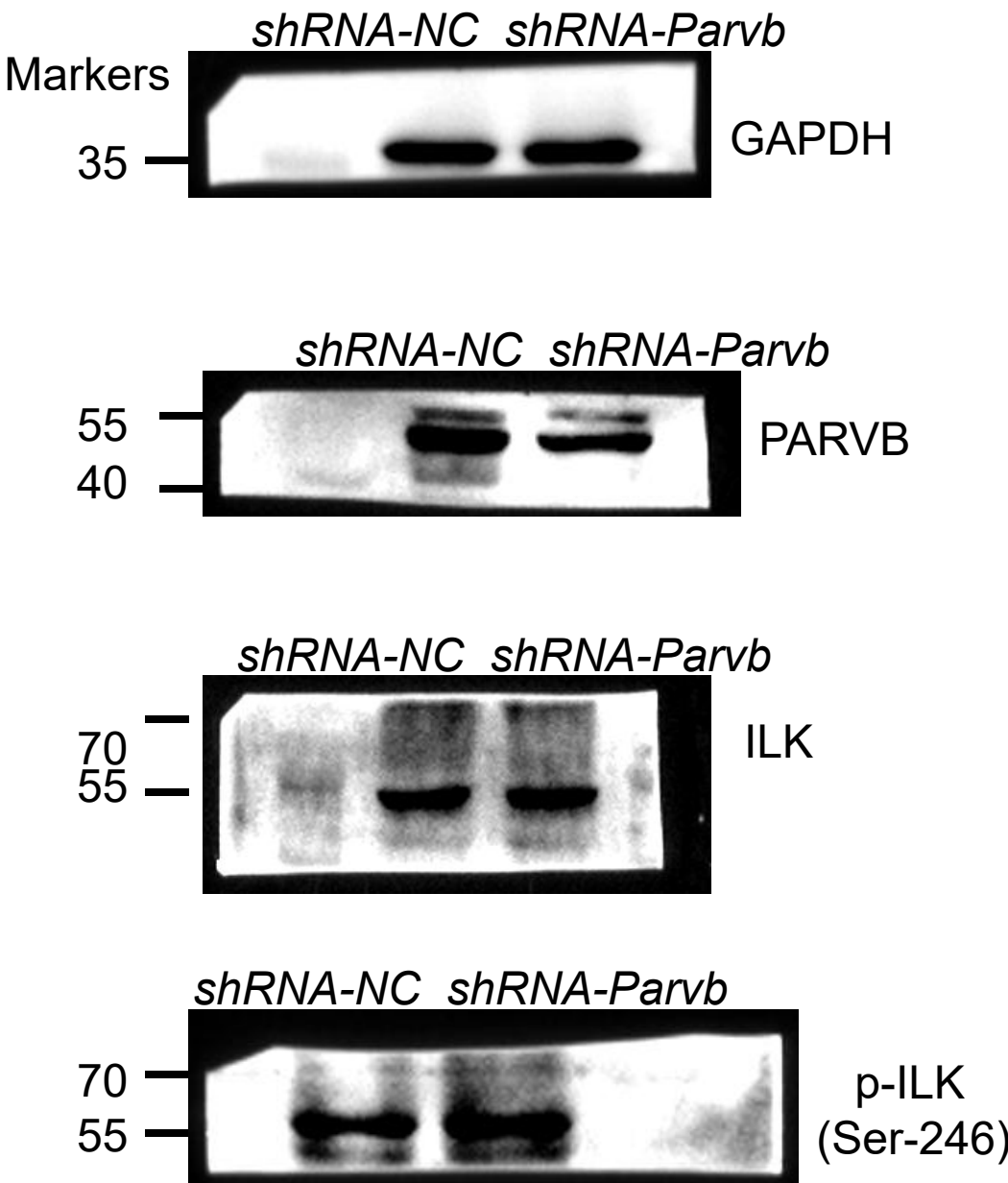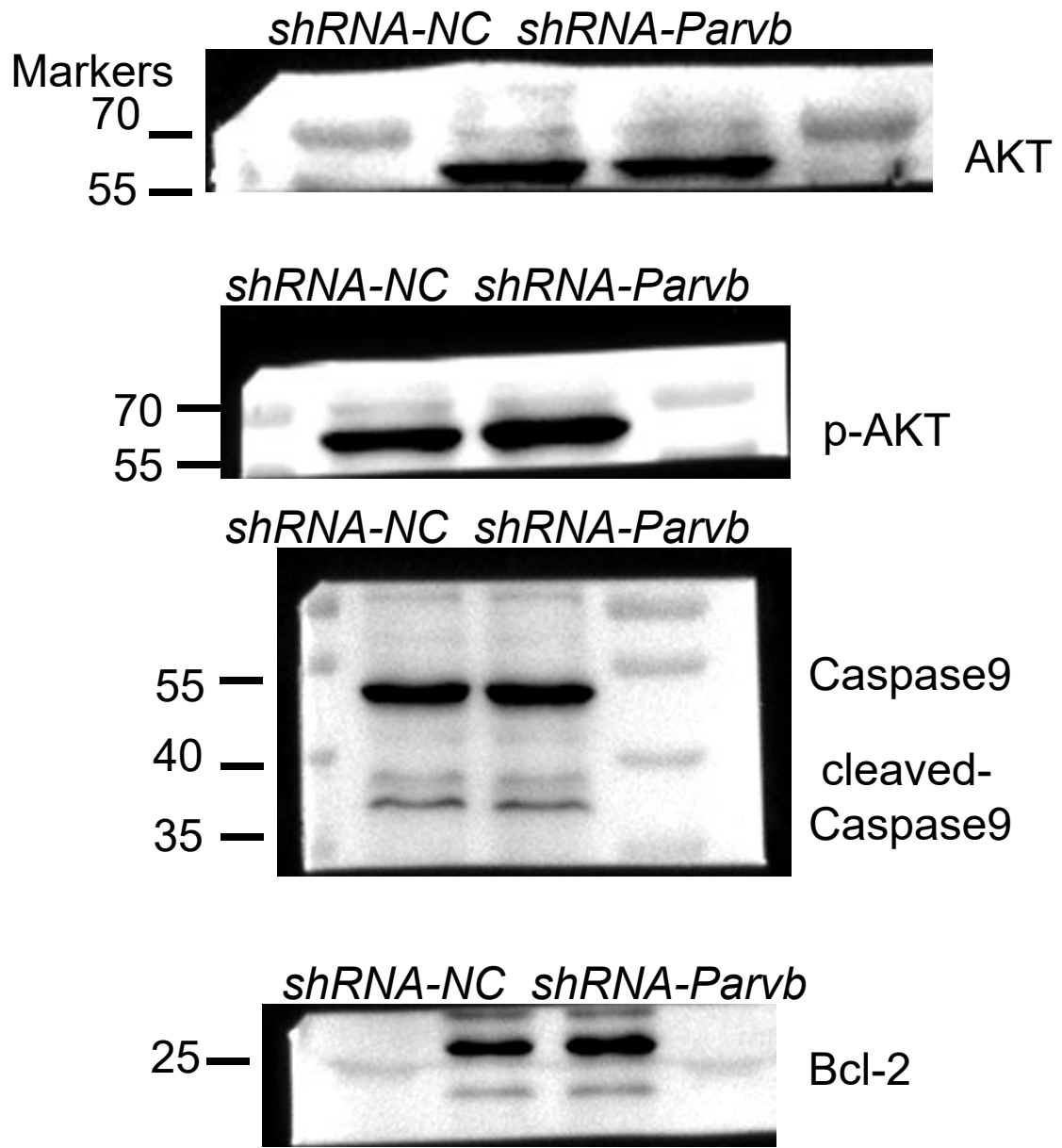

Fig. S7A

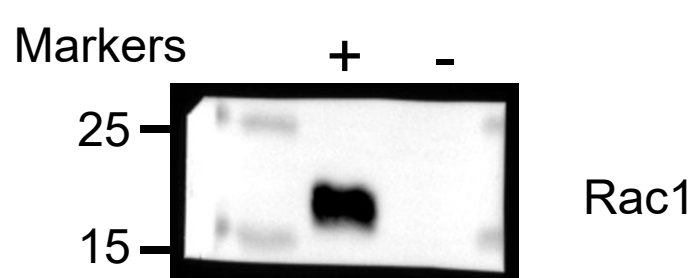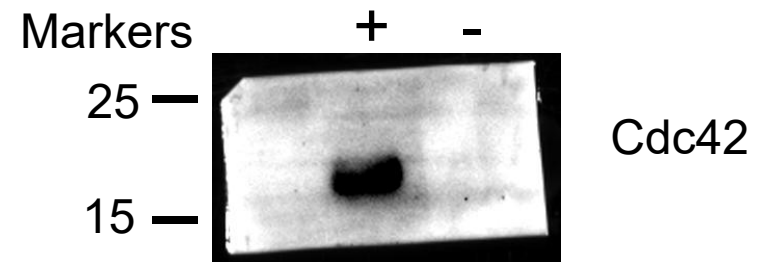

Fig. S7B

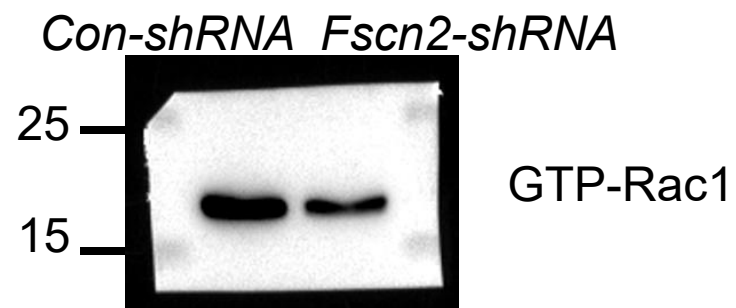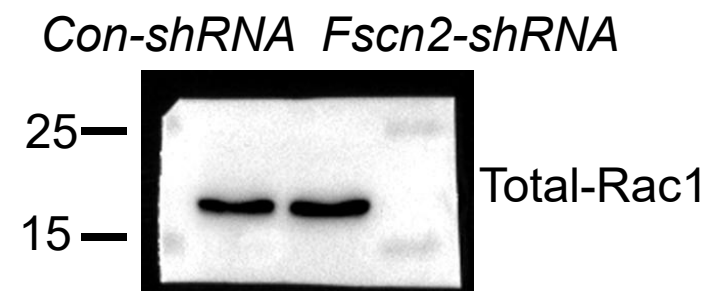

Fig. S7C

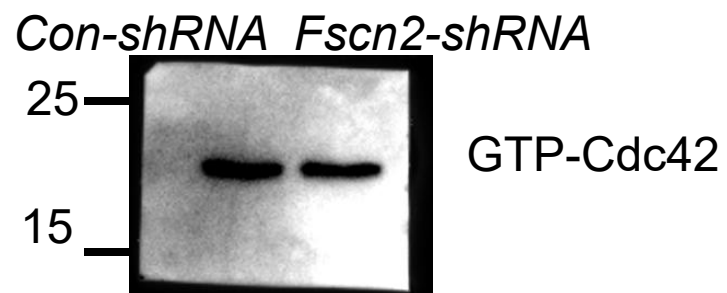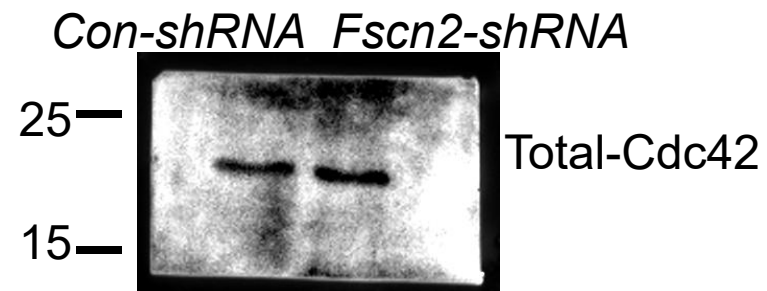

Fig. S8A

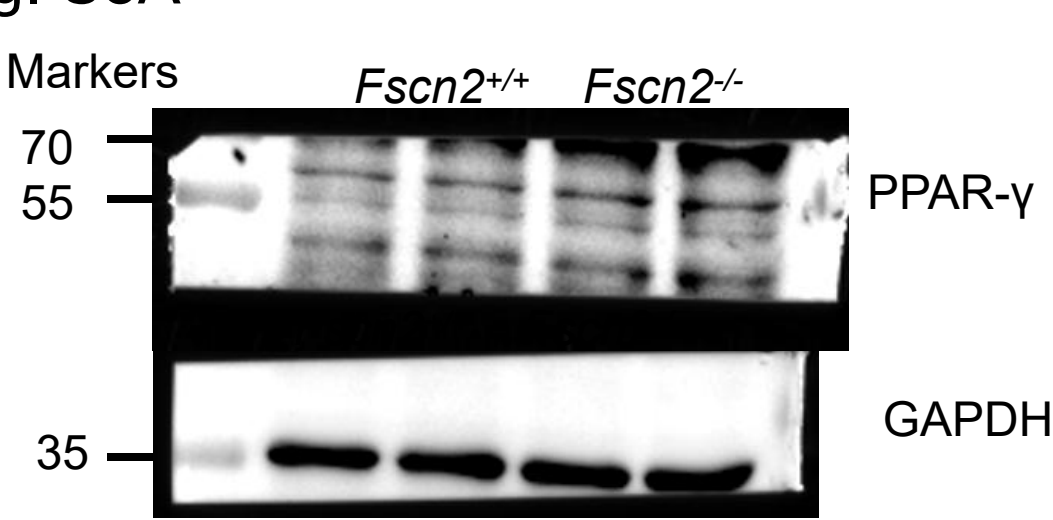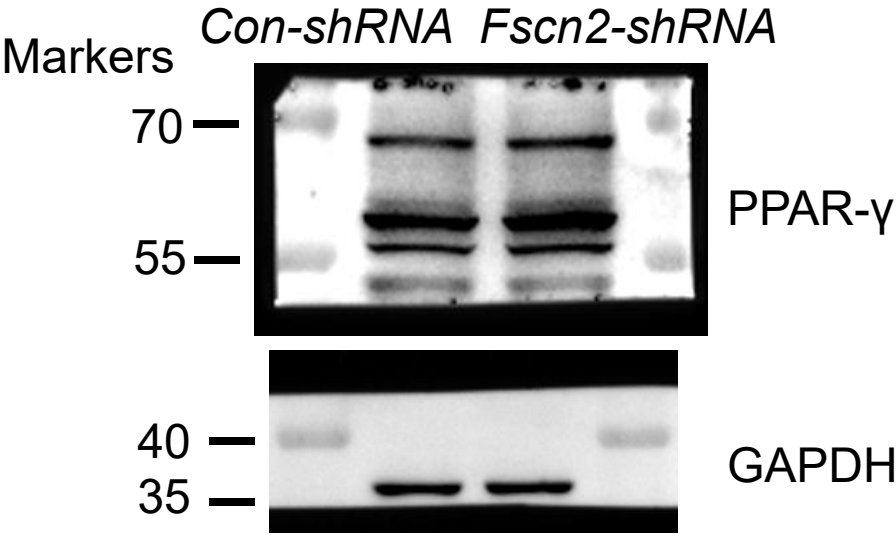

Fig. S8B

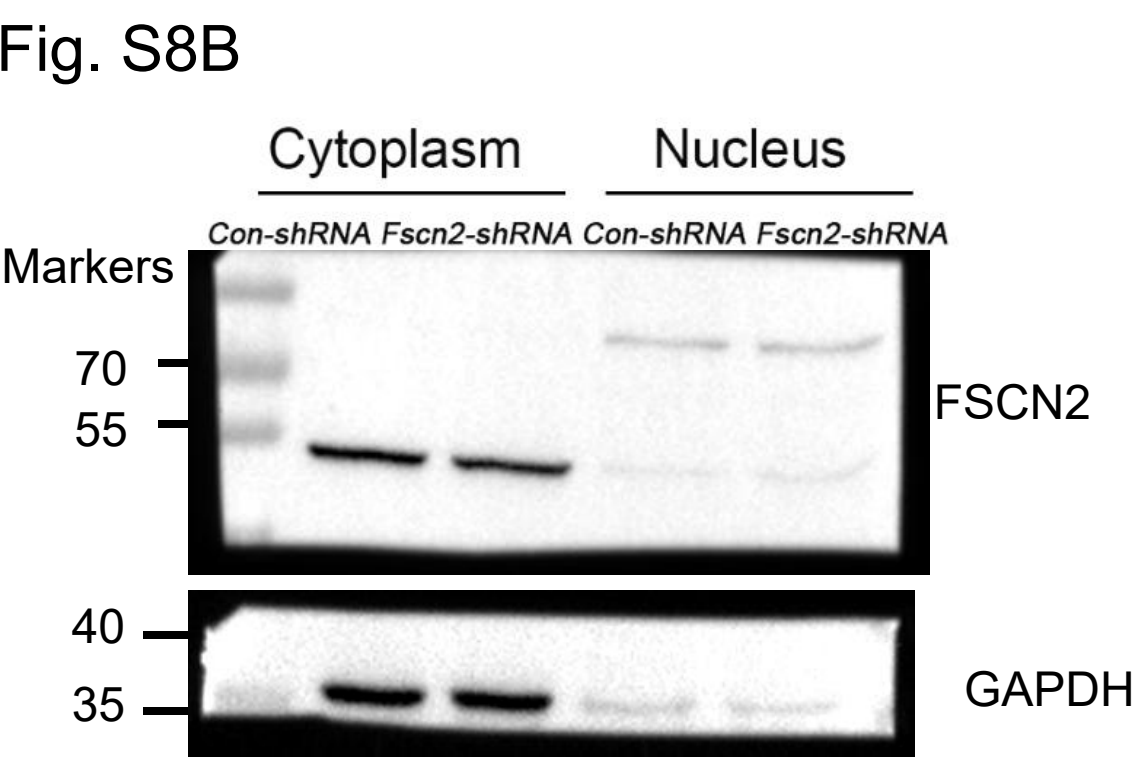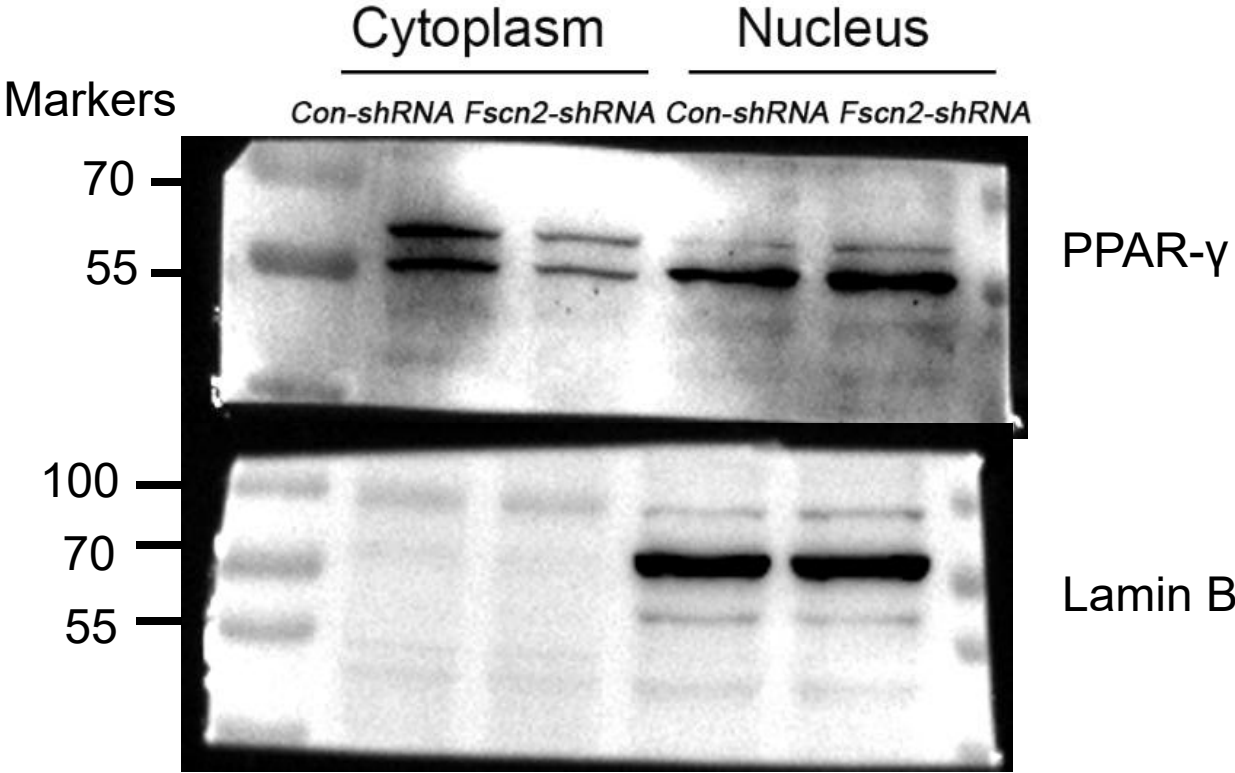

Fig. S8C

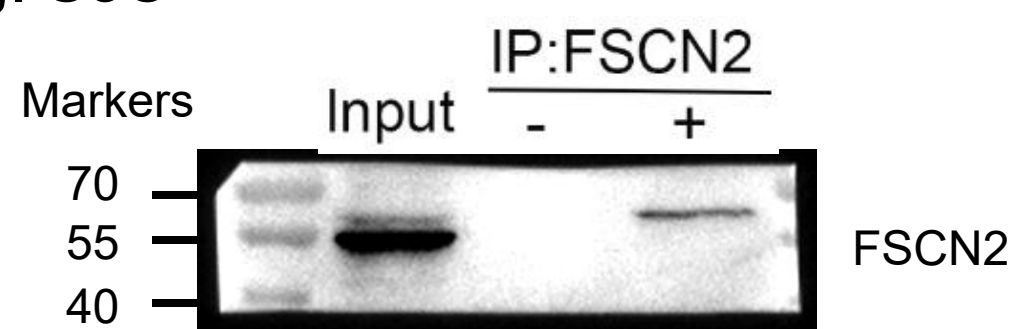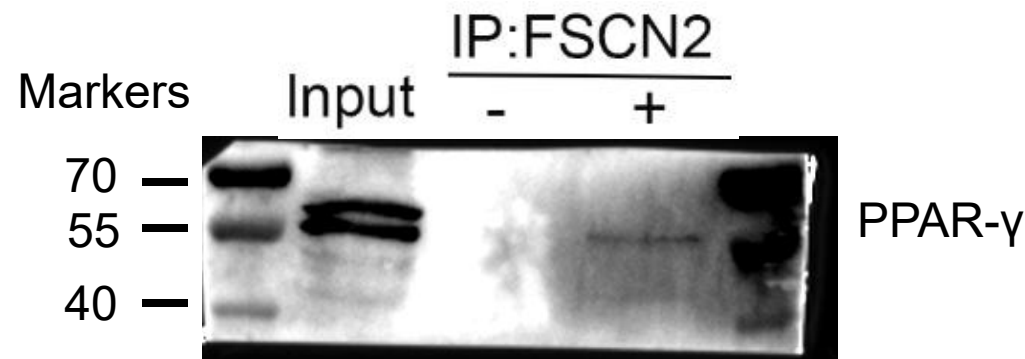

Fig. S8D

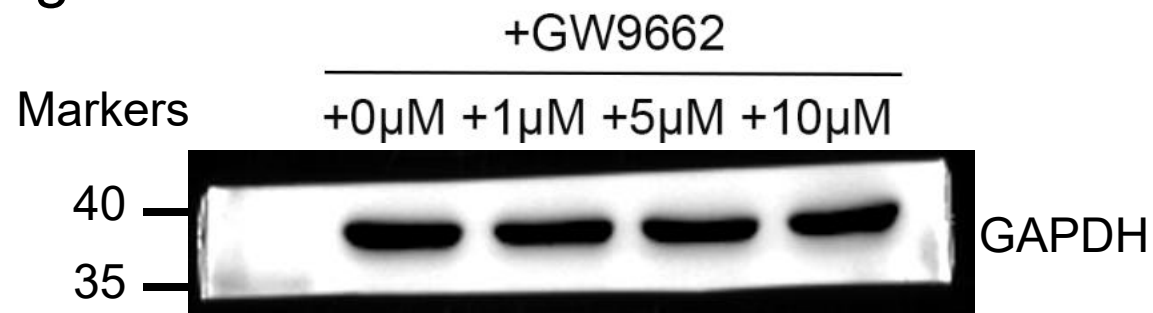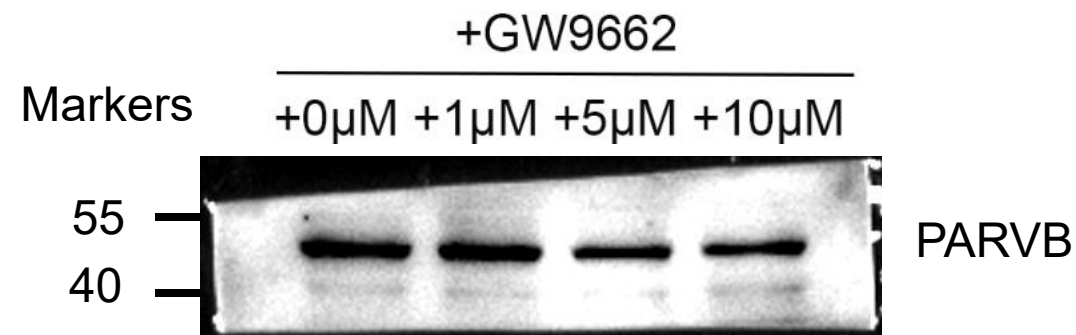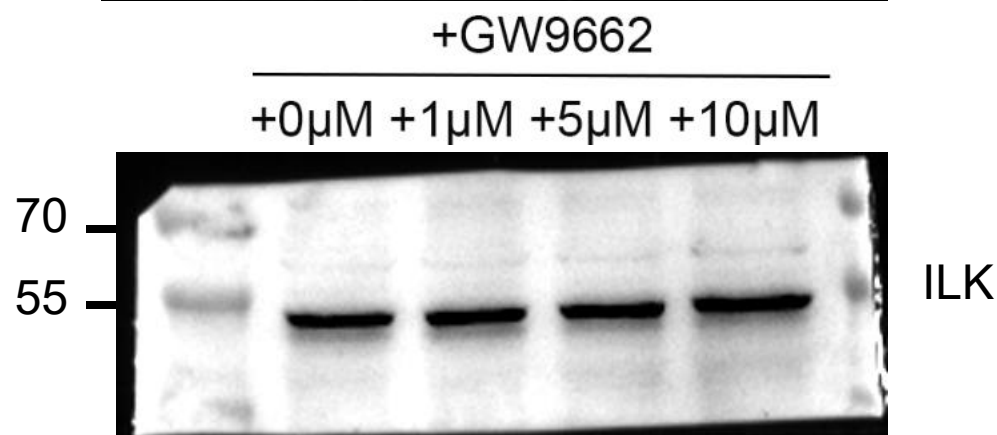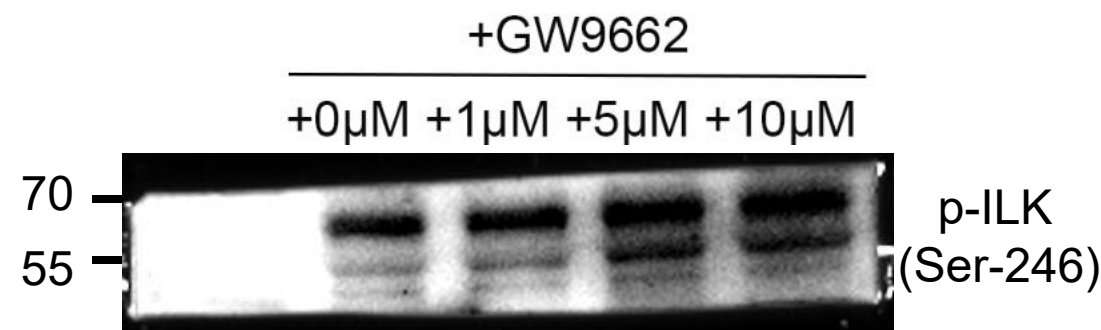

Fig. S8D

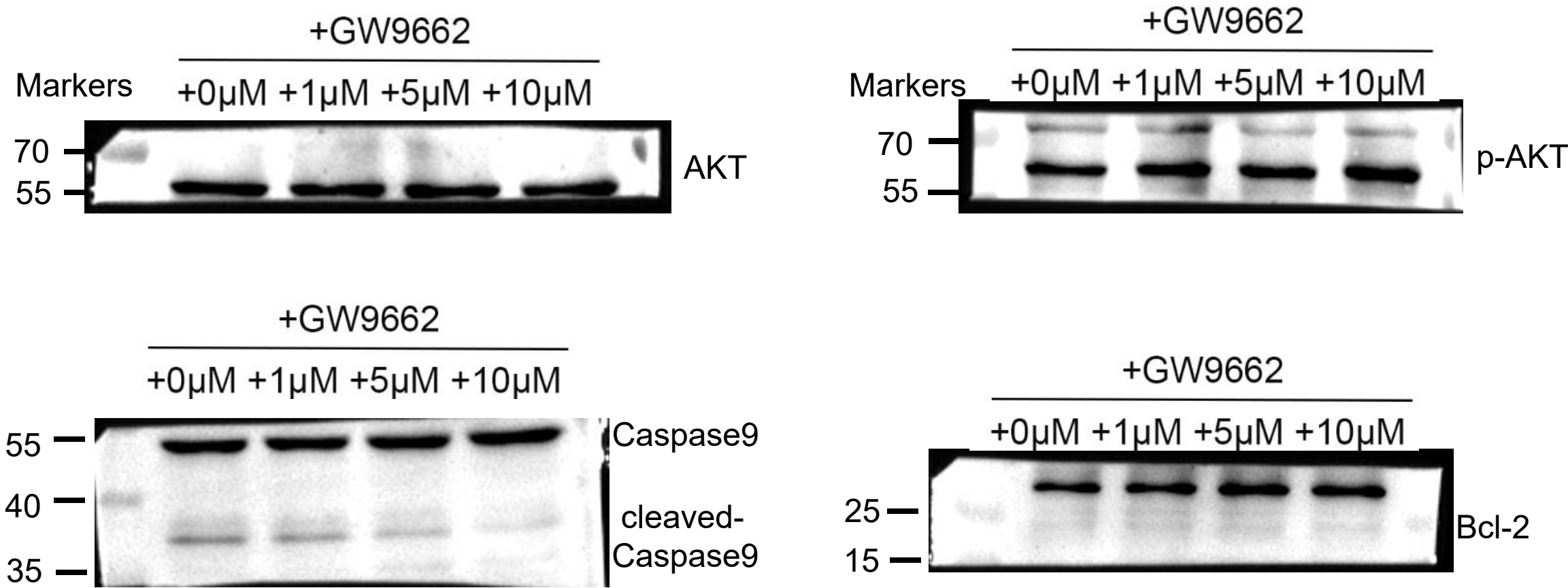

Supplement: Supplementary file 2 — Supplemental Material-2 [file 41420_2024_1851_MOESM2_ESM.pdf]
